# Mitochondrial inhibition enhances the sensitivity of pancreatic ductal adenocarcinoma cells to oncolytic adenovirus

**DOI:** 10.1016/j.omton.2026.201180

**Published:** 2026-03-18

**Authors:** Ryohei Shoji, Hiroshi Tazawa, Shinji Kuroda, Takeyoshi Nishiyama, Yoshinori Kajiwara, Motohiko Yamada, Yasuo Nagai, Hiroaki Inoue, Naoyuki Hashimoto, Satoru Kikuchi, Ryuichi Yoshida, Yuzo Umeda, Yasuo Urata, Shunsuke Kagawa, Toshiyoshi Fujiwara

**Affiliations:** 1 Department of Gastroenterological Surgery, Okayama University Graduate School of Medicine, Dentistry and Pharmaceutical Sciences, Okayama 700-8558, Japan; 2 Center for Innovative Clinical Medicine, Okayama University Hospital, Okayama 700-8558, Japan; 3 Department of HBP and Breast Surgery, Ehime University Graduate School of Medicine, Toon, Ehime 791-0295, Japan; 4 Oncolys BioPharma, Inc., Tokyo 105-0001, Japan

**Keywords:** MT: Regular Issue, pancreatic cancer, glycolysis, oncolytic virotherapy, CPI-613, PET/CT

## Abstract

The metabolism of cancer cells is associated with resistance to anticancer therapies. Pancreatic ductal adenocarcinoma (PDAC) cells exhibit glycolytic and non-glycolytic subtypes. Although oncolytic virotherapy is a novel antitumor modality, the relationship between metabolism and virus sensitivity remains unclear. We demonstrated the cytopathic activity of telomerase-specific, replication-competent oncolytic adenoviruses OBP-301 and p53-armed OBP-702 against PDAC cells. Here, we show the role of metabolism in the virus sensitivity of PDAC cells. The virus sensitivity of human PDAC cells of glycolytic (MIA PaCa-2, PK-45H) and non-glycolytic (PK-59, Capan-2) subtypes was assessed by evaluating replication, glycolysis, and glutamine metabolism through exposure to hypoxia and glucose deprivation or treatment with the mitochondrial metabolism inhibitor CPI-613. Glycolytic PDAC cells were sensitive, and non-glycolytic cells were resistant to oncolytic adenoviruses, which was improved by hypoxia and glucose deprivation or CPI-613 treatment to induce glycolytic activation. OBP-702-mediated p53 activation modulated glutamine metabolism to promote virus sensitivity. *In vivo* experiments demonstrated the antitumor efficacy of combination therapy with CPI-613 and OBP-702, and the utility of positron emission tomography/computed tomography metabolic parameters for assessing glycolytic activity. Our results suggest that non-glycolytic PDAC cells are refractory to oncolytic adenoviruses. CPI-613 is a promising reagent for overcoming virotherapy resistance in PDAC tumors.

## Graphical abstract

**Figure:**
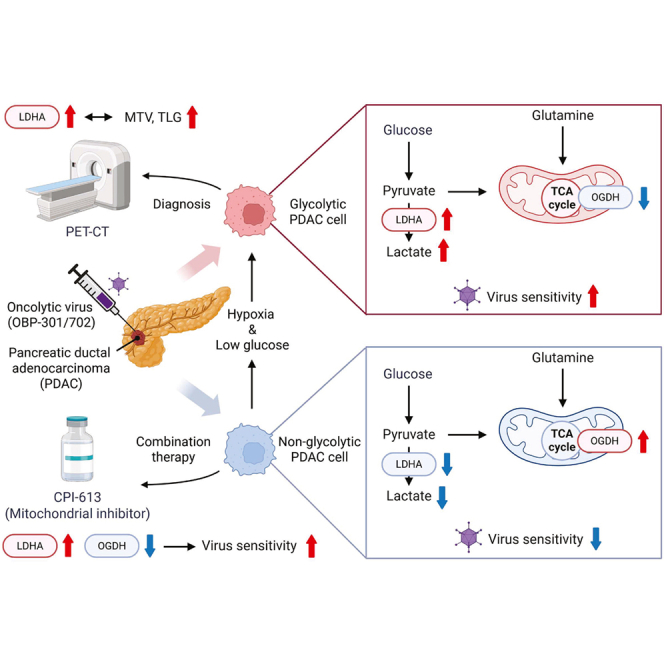

## Teaser

Pancreatic ductal adenocarcinoma (PDAC) cells exhibit glycolytic and non-glycolytic subtypes. Tazawa and colleagues demonstrate differences in sensitivity to oncolytic adenoviruses between glycolytic and non-glycolytic PDAC cells. The resistance of non-glycolytic cells to oncolytic adenoviruses was improved by treatment with a mitochondrial metabolism inhibitor.

## Introduction

Pancreatic ductal adenocarcinoma (PDAC) is the seventh leading cause of cancer-related death worldwide.^1^ Due to the advanced stage at diagnosis, 80%–90% of patients have unresectable PDAC tumors, and the 5-year survival rate is less than 10%.^1^ Multi-agent chemotherapy with gemcitabine, nab-paclitaxel, and FOLFIRINOX has demonstrated therapeutic efficacy for treating unresectable PDAC tumors and distant metastases.^2^ However, chemotherapy has not provided a greater survival benefit in patients with PDAC.^2^ Therefore, a novel therapeutic approach for eliminating PDAC cells is needed to improve patient clinical outcomes.

The metabolism of PDAC cells is closely associated with tumor invasion, metastasis, resistance to chemotherapy, and the acquisition of immune evasion characteristics.^2^ Several reports have shown that human PDAC cells can be classified into two metabolic subtypes, glycolytic and non-glycolytic, based on comprehensive metabolic gene profiling.^3^^,^^4^^,^^5^ Glycolytic cancer cells take up glucose and convert it into lactate without entry into the tricarboxylic acid (TCA) cycle in the mitochondria. Glycolytic activation produces only a small amount of energy in the form of ATP from one molecule of glucose, but the conversion to ATP is very rapid.^2^^,^^6^ Most cancer cells use the glycolytic pathway even under aerobic conditions, which is known as the Warburg effect.^7^ By contrast, non-glycolytic cancer cells produce a large amount of ATP via activation of the TCA cycle under aerobic conditions.^8^^,^^9^ Recent research has suggested that some cancer cells preferentially utilize glycolysis-independent energy metabolism.^3^^,^^10^ Given the hypovascularity of PDAC tumors, PDAC cells are frequently exposed to hypoxia and poor nutritional conditions, which lead to metabolic reprogramming that activates glycolysis as a means of adapting to the tumor microenvironment (TME).^11^^,^^12^

Oncolytic virotherapy has recently emerged as a novel therapeutic strategy to induce tumor-specific lytic cell death without affecting normal cells.^13^^,^^14^ We developed a telomerase-specific oncolytic adenovirus, OBP-301 (suratadenoturev), in which the human telomerase reverse transcriptase (hTERT) promoter drives the expression of the viral *E1A* and *E1B* genes (Figure S1A).^15^^,^^16^ OBP-301 can replicate selectively in tumor cells and induce tumor-specific lytic cell death with autophagy.^17^ To improve the therapeutic efficacy of OBP-301, we generated a modified OBP-301 variant (OBP-702) that induces the tumor suppressor *p53* gene by inserting the Egr1 promoter-driven p53 expression cassette into the E3 region of OBP-301 (Figure S1A).^18^^,^^19^ We demonstrated that OBP-702 exhibits more profound antitumor efficacy, with apoptosis and autophagy, compared with OBP-301 against human PDAC cells.^20^ *In vivo*, OBP-702 showed a significantly stronger antitumor effect against subcutaneous and orthotopic PDAC xenograft tumor models.^20^ However, the relationship between metabolic subtypes and sensitivity to oncolytic adenoviruses in PDAC cells remains unclear.

The metabolism of cancer cells is thought to be associated with their sensitivity to oncolytic adenoviruses. Human lung cancer A549 cells with a glycolytic phenotype are more sensitive to oncolytic adenoviruses than human ovarian cancer SKOV3 cells with a non-glycolytic phenotype.^21^ Oncolytic adenoviruses modulate glycolytic activity to create an optimal environment for promoting viral replication.^22^^,^^23^ Glutamine metabolism is also an important nutrient factor that can affect viral replication.^21^^,^^24^ The tumor suppressor gene p53 reportedly modulates glycolysis, glutamine metabolism, and mitochondrial activity, thereby potentially leading to enhanced adenoviral activity.^25^^,^^26^ These findings suggest that the therapeutic potential of oncolytic adenoviruses depends on the metabolic activity of target human cancer cells.

CPI-613 is a mitochondrial metabolism inhibitor that targets the TCA cycle by inhibiting pyruvate dehydrogenase and the α-ketoglutarate (α-KG) dehydrogenase complex, including 2-oxoglutarate dehydrogenase (OGDH).^27^ CPI-613 has been shown to suppress tumor growth in human PDAC xenograft tumor models.^28^ Several clinical trials of CPI-613 in combination with chemotherapy have been conducted in patients with locally advanced and metastatic PDAC (NCT01835041,^29^ NCT03435289, NCT03504423, NCT03699319). However, whether CPI-613 affects the therapeutic potential of oncolytic adenoviruses in PDAC cells remains to be elucidated.

In the present study, we investigated the therapeutic potential of the oncolytic adenoviruses OBP-301 and OBP-702 against human PDAC cells with different metabolic subtypes: a glycolytic subtype (MIA PaCa-2, PK-45H) and a non-glycolytic subtype (PK-59, Capan-2). The sensitivity of human PDAC cells to the oncolytic adenoviruses was further analyzed in terms of viral replication and cytopathic effect under normal or hypoxic conditions, as well as under glucose-depleted culture conditions, to modulate metabolic reprogramming. To develop a therapeutic strategy against virotherapy-resistant PDAC tumors, the therapeutic potential of combination therapy with OBP-702 and the mitochondrial metabolism inhibitor CPI-613 was analyzed using a subcutaneous PDAC tumor model. Moreover, to identify predictive biomarkers of the antitumor efficacy of oncolytic virotherapy, the diagnostic potential of positron emission tomography (PET)/computed tomography (CT) imaging was analyzed by calculating various metabolic parameters.

## Results

### Characterization of glycolytic activity in human PDAC cell lines

**Figure 1:**
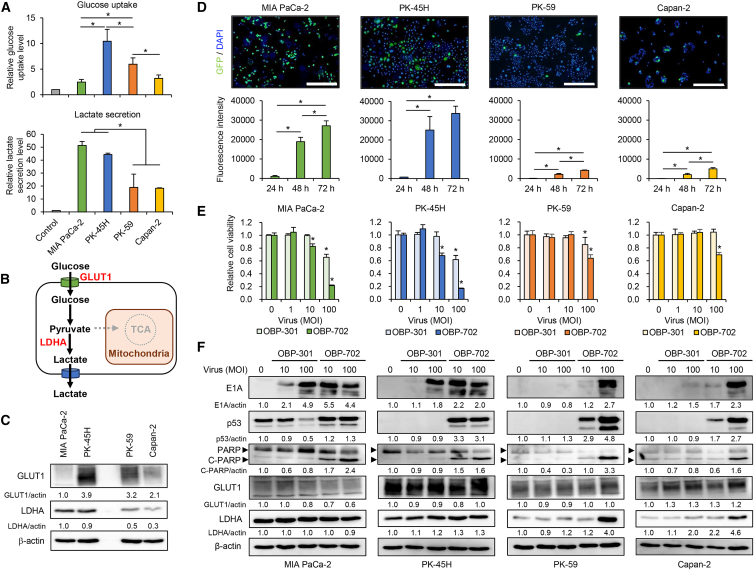
Comparison of metabolic characteristics and virus sensitivity between glycolytic and non-glycolytic PDAC cells (A) The upper graph shows glucose uptake in PDAC cells, presented as fold-increase compared with PBS, which was set as 1.0. The lower graph shows lactate secretion by PDAC cells, presented as fold-increase compared with PBS. Data are expressed as mean (SD) of independent experiment (*n* = 3). The statistical significance of differences among four groups was determined using one-way ANOVA followed by Turkey’s multiple comparison procedure. (B) Outline of glycolytic metabolism, shown from glucose uptake to lactate secretion. (C) Cell lysates of PDAC cells were subjected to western blot analysis for GLUT1 and LDHA. (D) PDAC cells were infected with OBP-401 at an MOI of 100 for 24, 48, or 72 h. The fluorescence intensity of GFP was analyzed under fluorescence microscopy (IX71; Olympus). Scale bars, 500 μm. Data are expressed as mean (SD) of independent experiment (*n* = 3). The statistical significance of differences among three groups was determined using one-way ANOVA followed by Turkey’s multiple comparison procedure. (E) PDAC cells were treated with OBP-301 or OBP-702 at the indicated MOIs for 72 h. Cell viability was quantified using the XTT assay and calculated relative to the mock-infected group. Data are expressed as mean (SD) of independent experiments (*n* = 5). The statistical significance of differences between two groups was determined using the Student’s *t* test. (F) PDAC cells were infected with OBP-301 or OBP-702 at the indicated MOIs for 48 h. Cell lysates were subjected to western blot analysis for E1A, p53, PARP, cleaved PARP (C-PARP), GLUT1, and LDHA. β-Actin was assayed as a loading control. The expression level of each protein was calculated relative to that of MIAPaCa-2 cells or mock-treated cells, which was set at 1.0. ∗, *p* < 0.05.

Several reports of comprehensive metabolic gene profiling studies have identified glycolytic and non-glycolytic subtypes in human PDAC cell lines.^3^^,^^4^^,^^5^ To evaluate the therapeutic potential of oncolytic adenoviruses in human PDAC cells with different metabolic subtypes, we used human PDAC cells showing a glycolytic subtype (MIA PaCa-2, PK-45H) and non-glycolytic subtype (PK-59, Capan-2) according to previous reports.^3^^,^^4^^,^^5^ To evaluate the glycolytic activity of these cells, glucose uptake and lactate secretion were analyzed by metabolic assays. Glucose uptake was higher in glycolytic PK-45H and non-glycolytic PK-59 cells compared with other cells (Figure 1A). Lactate secretion was higher in glycolytic PDAC cells compared with non-glycolytic cells (Figure 1A). Next, we analyzed the expression of glycolysis-related proteins, glucose transporter 1 (GLUT1) (a glucose transporter for glucose uptake) and lactate dehydrogenase A (LDHA) (an enzyme that catalyzes the conversion of pyruvate to lactate), by western blotting (Figure 1B). The expression of GLUT1 was higher in glycolytic PK-45H and non-glycolytic PK-59 cells, consistent with the glucose uptake results (Figure 1C). The expression of LDHA was higher in glycolytic PDAC cells compared with non-glycolytic cells, consistent with the lactate secretion results (Figure 1C). These results suggest that glycolytic PDAC cells have the potential to secrete more lactate compared with non-glycolytic cells via LDHA activation.

### Glycolytic PDAC cells are more sensitive to oncolytic adenoviruses than non-glycolytic cells

To investigate the relationship between virus sensitivity and glycolysis in PDAC cells, PDAC cells were infected with green fluorescent protein (GFP)-expressing OBP-401 (Figure S1A), which enables the confirmation of viral infection and replication in target cells by evaluating GFP expression.^30^ Glycolytic PDAC cells showed significantly higher GFP expression than non-glycolytic cells (Figure 1D), suggesting that glycolytic PDAC cells are more sensitive to virus infection. To confirm this result, PDAC cells were infected with OBP-301 and p53-armed OBP-702 (Figure S1A). An XTT assay demonstrated that glycolytic PDAC cells were more sensitive to OBP-301 and OBP-702 than non-glycolytic cells (Figure 1E). Western blot analysis showed that, in association with decreased cell viability, OBP-301 (100 MOI) increased the expression of E1A protein and decreased the expression of mutant p53 protein in glycolytic PDAC cells (Figure 1F). The expression of cleaved poly(ADP-ribose) polymerase (PARP) protein (an apoptosis marker) was not increased by OBP-301. OBP-702 (10 and 100 MOIs) increased the expression of E1A, p53, and cleaved PARP proteins in glycolytic PDAC cells (Figure 1F). In contrast, non-glycolytic PDAC cells showed increased expression of E1A, p53, and cleaved PARP proteins when infected with OBP-702 (100 MOI) (Figure 1F). OBP-301 (100 MOI) slightly increased the expression of E1A protein in non-glycolytic Capan-2 cells (Figure 1F). Real-time PCR analysis demonstrated that OBP-301 and OBP-702 increased the expression of E1A DNA and p53 mRNA in glycolytic PDAC cells more strongly than in non-glycolytic cells (Figure S2). Although OBP-702 increased the expression of the GLUT1 and LDHA proteins in non-glycolytic PDAC cells (Figure 1F), lactate secretion was not affected by OBP-702 in glycolytic and non-glycolytic PDAC cells (Figure S3). These results suggest that glycolytic PDAC cells are more sensitive to oncolytic adenoviruses than non-glycolytic cells.

To exclude the possibility that high virus sensitivity depends on the expression of hTERT and adenovirus receptors, and the proliferative capacity of glycolytic PDAC cells, PDAC cells were analyzed by PCR, fluorescence-activated cell sorting (FACS) analyses, and an XTT assay. Glycolytic PDAC cells exhibited lower expression of *hTERT* mRNA than non-glycolytic cells (Figure S4A). The expression of coxsackievirus and adenovirus receptor (CAR) and integrins αVβ3 and αVβ5, as well as the proliferation rate, varied among PDAC cell lines (Figures S4B and S4C). These results suggest that glycolytic PDAC cells exhibit high virus sensitivity independent of hTERT expression, adenovirus receptors, and proliferation capacity.

### The glycolytic pathway is important for maintaining virus sensitivity in PDAC cells

**Figure 2:**
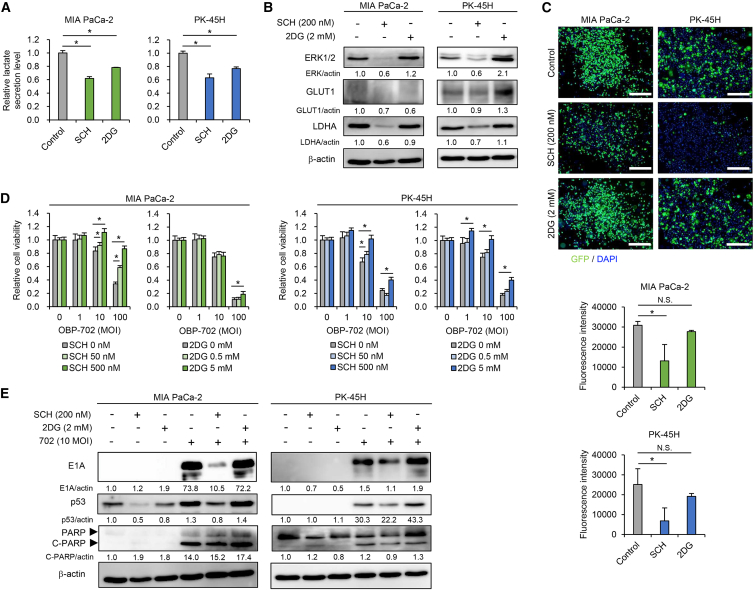
Glycolysis inhibitors diminish the virus sensitivity of glycolytic PDAC cells MIA PaCa-2 and PK-59 cells were treated with SCH772984 (SCH) (200 nM) or 2DG (2 mM), followed by infection with OBP-401 (100 MOI) or OBP-702 (10 MOI). (A) Lactate secretion by MIA PaCa-2 and PK-45H cells treated with SCH772984 or 2DG, presented as fold-increase compared with the control group, which was set as 1.0. Data are expressed as mean (SD) of independent experiments (*n* = 3). The statistical significance of differences between two groups was determined using the Student’s *t* test. (B) Cell lysates of MIA PaCa-2 and PK-45H cells treated with SCH or 2DG for 48 h were subjected to western blot analysis for ERK1/2, GLUT1, and LDHA. (C) MIA PaCa-2 and PK-45H cells were treated with SCH or 2DG, followed by infection with OBP-401 (100 MOI) for 24 or 48 h. Upper panels show representative photographs of immunocytochemical staining for GFP in each group 48 h after infection. Scale bars, 500 μm. Lower graphs show the fluorescence intensity of GFP analyzed under fluorescence microscopy. Data are expressed as mean (SD) of independent experiment (*n* = 3). The statistical significance of differences between two groups was determined using the Student’s *t* test. (D) MIA PaCa-2 and PK-45H cells were co-treated with OBP-702 and SCH772984 or 2DG at the indicated dose for 72 h. Cell viability was quantified using the XTT assay and calculated relative to the mock-infected group. Data are expressed as mean (SD) of independent experiment (*n* = 5). The statistical significance of differences between two groups was determined using the Student’s *t* test. (E) Cell lysates of MIA PaCa-2 and PK-45H cells co-treated with SCH or 2DG and OBP-702 (10 MOI) for 48 h were subjected to western blot analysis for E1A, p53, PARP, and cleaved C-PARP. β-actin was assayed as a loading control. The expression level of each protein was calculated relative to that of mock-treated cells, which was set at 1.0. N.S., not significant; ∗, *p* < 0.05.

To evaluate the role of glycolysis in the virus sensitivity of PDAC cells, glycolytic PDAC cells were treated with two glycolysis inhibitors, SCH772984 and 2-deoxy-D-glucose (2DG). SCH772984 is an inhibitor of the extracellular signal-regulated kinase (ERK) signaling pathway, which is upstream of glycolysis.^31^ 2DG is a synthetic glucose analog that competitively inhibits glucose uptake.^32^ We analyzed the viability of glycolytic PDAC cells at 24 and 72 h after treatment with SCH772984 and 2DG using the XTT assay (Figure S5). SCH772984 (>500 nM) or 2DG (>10 mM) significantly suppressed the viability of glycolytic PDAC cells at 24 h after treatment, whereas SCH772984 (>50 nM) or 2DG (>1 mM) significantly reduced the viability of glycolytic PDAC cells at 72 h after treatment (Figure S5). Administration of SCH772984 (200 nM) and 2DG (2 mM) significantly inhibited lactate secretion in glycolytic PDAC cells (Figure 2A), while the viability of glycolytic PDAC cells was maintained at 24 h after treatment (Figure S5). SCH772984 decreased the expression of ERK1/2, GLUT1, and LDHA proteins in glycolytic PDAC cells, whereas 2DG conversely increased the expression of ERK1/2 protein in glycolytic PDAC cells and GLUT1 protein in PK-45H cells (Figure 2B). These results suggest that SHC772984 suppresses glycolysis in PDAC cells, and 2DG is insufficient to inhibit glycolysis in PDAC cells.

We then analyzed the effect of SCH772984 and 2DG on the virus sensitivity of glycolytic PDAC cells. SCH772984 significantly suppressed OBP-401-mediated GFP expression and OBP-702–mediated cytopathic activity (Figures 2C and 2D). OBP-702–mediated upregulation of E1A, p53, and cleaved PARP protein expression was also suppressed by SCH772984 (Figure 2E). By contrast, 2DG significantly suppressed only OBP-702–mediated cytopathic activity (Figure 2D) but not OBP-401-mediated GFP expression (Figure 2C). OBP-702-mediated upregulation of E1A, p53, and cleaved PARP protein was not suppressed by 2DG (Figure 2E). These results suggest that the ERK-related glycolytic pathway plays a crucial role in the virus sensitivity of PDAC cells, whereas 2DG-mediated suppression of glucose uptake is insufficient to inhibit glycolysis in PDAC cells.

### OBP-702 modulates glutamine metabolism by p53 activation in PDAC cells expressing OGDH

**Figure 3:**
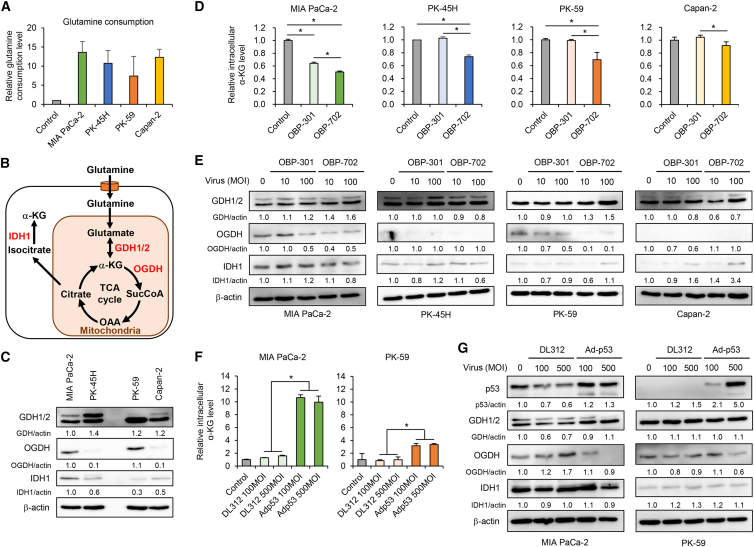
p53 activation modulates glutamine metabolism in PDAC cells (A) Glutamine consumption in PDAC cells, presented as fold-increase compared with PBS, which was set as 1.0. (B) Outline of glutamine metabolism, shown from glutamine uptake to α-KG production. (C) Lysates of PDAC cells were subjected to western blot analysis for GDH1/2, OGDH, and IDH1. (D) PDAC cells were infected with OBP-301 or OBP-702 at an MOI of 100 for 48 h. The amount of intracellular α-KG in PDAC cells is shown as fold-increase compared with the mock-infected group, which was set as 1.0. Data are expressed as mean (SD) of independent experiments (*n* = 3). The statistical significance of differences between two groups was determined using the Student’s *t* test. (E) PDAC cells were infected with OBP-301 or OBP-702 at the indicated MOIs for 72 h. Cell lysates were subjected to western blot analysis for GDH1/2, OGDH, and IDH1. (F) MIA PaCa-2 and PK-59 cells were infected with DL312 or Adp53 at the indicated MOIs for 24 h. The amount of intracellular αKG in PDAC cells is presented as fold-increase compared with mock-infected control groups. Data are expressed as mean (SD) of independent experiments (*n* = 3). The statistical significance of differences among four groups was determined using one-way ANOVA followed by Turkey’s multiple comparison procedure. (G) MIA PaCa-2 and PK-59 cells were infected with DL312 or Adp53 at the indicated MOIs for 48 h. Cell lysates were subjected to western blot analysis for p53, GDH1/2, OGDH, and IDH1. β-Actin was assayed as a loading control. The expression level of each protein was calculated relative to that of MIAPaCa-2 cells or mock-treated cells, which was set at 1.0. ∗, *p* < 0.05.

Glutamine metabolism has been shown to affect adenovirus replication.^21^^,^^24^ We therefore evaluated glutamine metabolism in PDAC cells using a metabolic assay of glutamine consumption. However, no significant difference in glutamine consumption was observed between glycolytic and non-glycolytic PDAC cells (Figure 3A). We then used western blotting to analyze the expression of glutamine metabolism–related proteins, glutamate dehydrogenase 1/2 (GDH1/2) (enzymes that catalyze the reversible conversion of glutamate to α-KG), OGDH (an enzyme that catalyzes the conversion of α-KG to succinyl-CoA in the TCA cycle), and isocitrate dehydrogenase 1 (IDH1) (an enzyme that catalyzes the conversion of isocitrate to α-KG in the cytoplasm) (Figure 3B). Expression of GDH1/2 was observed in all PDAC cells, whereas expression of OGDH was detected in glycolytic MIA PaCa-2 and non-glycolytic PK-59 cells (Figure 3C). Expression of IDH1 was higher in glycolytic PDAC cells compared with non-glycolytic PDAC cells (Figure 3C). These results suggest that glutamine metabolism varies between glycolytic and non-glycolytic PDAC cells.

The tumor suppressor p53 has been shown to activate glutamine metabolism in cancer cells.^33^ We therefore evaluated whether p53-armed OBP-702 affects glutamine metabolism in PDAC cells using a metabolic assay for intracellular α-KG, an intermediate produced during glutamine metabolism that is necessary for viral genome synthesis.^21^ OBP-702 significantly decreased the intracellular level of α-KG in PDAC cells more strongly than OBP-301 (Figure 3D). By contrast, glutamine consumption was not affected by infection with OBP-301 or OBP-702 (Figure S6). These results suggest that OBP-702 modulates glutamine metabolism in PDAC cells.

Next, we used western blotting to analyze the expression of GDH1/2, OGDH, and IDH1 in PDAC cells infected with OBP-301 or OBP-702. The expression of GDH1/2 was increased in glycolytic MIAPaCa-2 and non-glycolytic PK-59 cells by OBP-702 but not OBP-301 (Figure 3E). The expression of OGDH was detected in glycolytic MIAPaCa-2 and non-glycolytic PK-59 cells, and OBP-702 decreased the expression of OGDH protein more strongly than OBP-301 (Figure 3E). By contrast, the expression of IDH1 was decreased in glycolytic PDAC cells and increased in non-glycolytic PDAC cells after OBP-702 treatment (Figure 3E). These results suggest that OBP-702 modulates glutamine metabolism-related proteins in MIAPaCa-2 and PK-59 cells expressing OGDH more strongly than in other cells without OGDH expression.

To evaluate whether glutamine deprivation affects the virus sensitivity of glycolytic PDAC cells, MIAPaCa-2 and PK-45H cells were infected with OBP-301 or OBP-702 under culture conditions with or without glutamine supplementation (Figure S7). Western blot analysis showed that glutamine restriction suppressed virus-mediated E1A accumulation in MIAPaCa-2 cells and, conversely, enhanced it in PK-45H cells. The expression of p53 was associated with E1A expression in OBP-702-infected PDAC cells. Glutamine depletion increased cleaved PARP expression in MIAPaCa-2 cells treated with or without virus infection, whereas cleaved PARP expression was increased in OBP–702-infected PK-45H cells. To investigate the relationship between virus sensitivity and glutamine deprivation in glycolytic PDAC cells, MIAPaCa-2 and PK-45H cells were infected with GFP-expressing OBP-401 (Figure S8). Glutamine restriction significantly suppressed OBP-401-mediated GFP expression in MIAPaCa-2 cells but not in PK-45H cells. These results suggest that glutamine is necessary for the survival and virus sensitivity of MIAPaCa-2 cells expressing OGDH but not PK-45H cells without OGDH expression.

To evaluate the role of p53 in the modulation of glutamine metabolism in PDAC cells expressing OGDH, we used the p53-expressing replication-defective adenovirus Ad-p53 and the non-expressing replication-defective adenovirus DL312 (Figure S1B). Glycolytic MIA PaCa-2 and non-glycolytic PK-59 cells expressing OGDH were treated with the p53-expressing adenovirus Ad-p53 and the non-expressing control adenovirus DL312. Ad-p53 significantly increased the intracellular α-KG level in both PDAC cell types (Figure 3F), whereas neither glutamine consumption nor lactate secretion was affected by Ad-p53 (Figure S9). Ad-p53 decreased OGDH expression, whereas the expression of GDH1/2 and IDH1 was not affected by Ad-p53 (Figure 3G). To further evaluate the role of p53 in virus sensitivity, PDAC cells were infected with Ad-p53 or DL312, followed by infection with GFP-expressing OBP-401. Ad-p53 significantly increased OBP-401-mediated GFP expression compared with the control DL312 (Figure S10). These results suggest that OBP-702 modulates glutamine metabolism by p53 activation to promote virus sensitivity in PDAC cells expressing OGDH.

### Glycolytic PDAC tumors maintain high sensitivity to oncolytic adenoviruses

**Figure 4:**
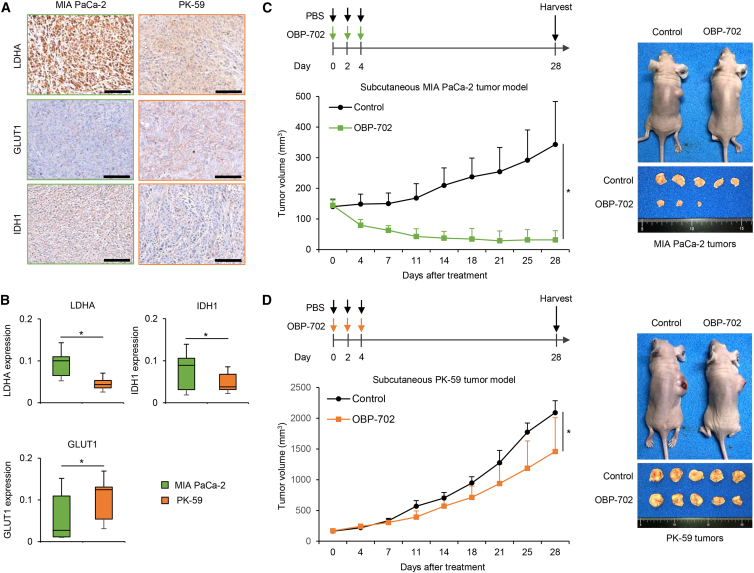
Comparison of metabolic phenotypes and virus sensitivity in subcutaneous tumor models with glycolytic and non-glycolytic PDAC cells (A) Representative photographs of immunohistochemical staining for LDHA, GLUT1, and IDH1 in each group. Scale bars, 100 μm. (B) Expression levels of LDHA, GLUT1, and IDH1, calculated by dividing the DAB intensity by the number of cells in randomly selected fields. Data are expressed as mean (SD) of independent experiments (*n* = 5). The statistical significance of differences between two groups was determined using the Student’s *t* test. (C) MIA PaCa-2 tumor-bearing mice received intratumoral injections of PBS (black arrows) or OBP-702 (green arrows) every other day for 3 cycles. Data are expressed as mean (SD) of independent experiments (*n* = 5). The statistical significance of differences between two groups was determined using the Student’s *t* test. (D) PK-59 tumor-bearing mice received intratumoral injections of PBS (black arrows) or OBP-702 (orange arrows). The upper right photographs show tumor-bearing mice in the control and OBP-702-treated groups. The lower right photographs show tumors in the mock and OBP-702 groups. Data are expressed as mean (SD) of independent experiments (*n* = 5). The statistical significance of differences between two groups was determined using the Student’s *t* test. ∗, *p* < 0.05.

To investigate whether PDAC cells maintain their metabolic phenotype in developing tumors, we obtained subcutaneous PDAC tumors with glycolytic MIA PaCa-2 and non-glycolytic PK-59 cells at similar tumor sizes (approximately 10 mm in diameter) and analyzed the expression of LDHA, GLUT1, and IDH1 in tumor tissues by immunohistochemistry. Glycolytic MIA PaCa-2 tumors showed significantly higher expression of LDHA and IDH1 and lower expression of GLUT1 compared with non-glycolytic PK-59 tumors (Figures 4A and 4B), in agreement with *in vitro* experiments (Figures 1 and 3). These results suggest that glycolytic and non-glycolytic PDAC tumors maintain their metabolic phenotypes.

To evaluate the therapeutic potential of OBP-702 against PDAC tumors, we then examined subcutaneous tumor models with glycolytic MIA PaCa-2 and non-glycolytic PK-59 cells. PDAC tumor-bearing mice received intratumoral injection of OBP-702 or PBS every other day for 3 cycles (Figures 4C and 4D). OBP-702 significantly suppressed the growth of glycolytic MIA PaCa-2 tumors more strongly than that of non-glycolytic PK-59 tumors (Figures 4C and 4D). Moreover, 2 of 5 MIA PaCa-2 tumors completely disappeared after treatment with OBP-702 (Figure 4C). These results suggest that glycolytic PDAC tumors are more sensitive to OBP-702 than non-glycolytic tumors.

### Hypoxia and glucose deprivation promote glycolysis in non-glycolytic PDAC cells

**Figure 5:**
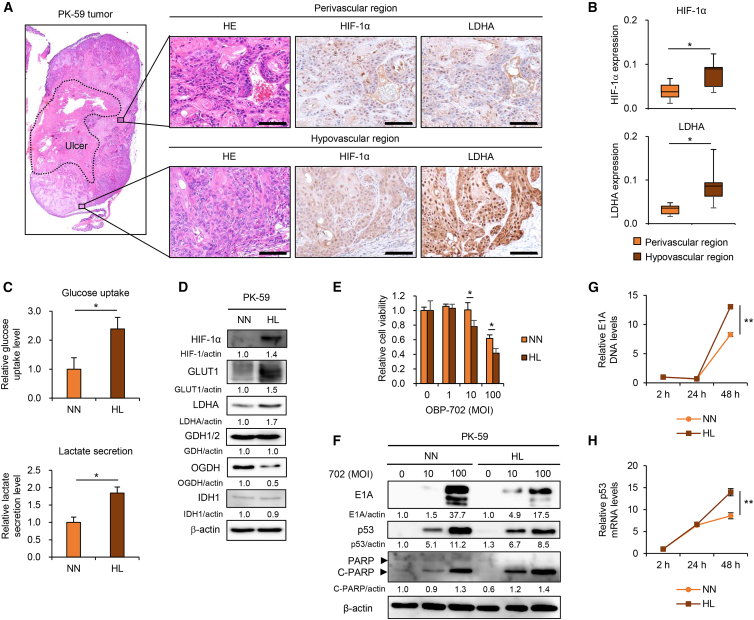
Hypoxia and glucose deprivation enhance virus sensitivity in non-glycolytic PK-59 cells (A) Representative photographs of H&E staining or immunohistochemical staining for LDHA and HIF-1α in each area of a PK-59 tumor. Scale bars, 100 μm. (B) Expression of HIF-1α and LDHA was calculated by dividing the DAB intensity by the number of cells in randomly selected fields. Data are expressed as mean (SD) of independent experiments (*n* = 5). The statistical significance of differences between two groups was determined using the Student’s *t* test. (C) The upper graph shows glucose uptake in PDAC cells under hypoxia and low-glucose (HL) culture conditions, presented as fold-increase compared with the normal (NN) culture condition, which was set as 1.0. The lower graph shows lactate secretion by PDAC cells under HL culture conditions, presented as fold-increase compared with the NN culture condition, which was set as 1.0. Data are expressed as mean (SD) of independent experiments (*n* = 3). The statistical significance of differences between two groups was determined using the Student’s *t* test. (D) Lysates of PK-59 cells cultured under HL and NN conditions were subjected to western blot analysis for HIF-1α, GLUT1, LDHA, GDH1/2, OGDH, and IDH1. (E) PK-59 cells were infected with OBP-702 at the indicated MOIs for 72 h under HL and NN culture conditions. Cell viability was quantified using the XTT assay and calculated relative to the mock-infected group. Data are expressed as mean (SD) of independent experiments (*n* = 5). The statistical significance of differences between two groups was determined using the Student’s *t* test. (F) Cell lysates were subjected to western blot analysis for E1A, p53, PARP, and cleaved C-PARP. β-Actin was assayed as a loading control. The expression level of each protein was calculated relative to that of the NN group or mock-treated cells, which was set at 1.0. (G and H) Copy number of adenoviral E1A DNA (G) and expression of p53 mRNA (B) were analyzed by real-time RT-PCR. The relative copy number of E1A DNA and relative expression of p53 mRNA are presented as fold-increase compared with 2 h, which was set as 1.0. Data are expressed as mean (SD) of independent experiments (*n* = 3). The statistical significance of differences between two groups was determined using the Student’s *t* test. ∗, *p* < 0.05; ∗∗, *p* < 0.01.

The metabolism of PDAC cells is affected not only by intrinsic genetic factors but also by extrinsic TME factors, such as hypoxia and glucose deprivation.^12^^,^^34^ Therefore, we identified the most important factors involved in promoting glycolysis in non-glycolytic PDAC tumors. The expression of LDHA between the perivascular and hypovascular regions in non-glycolytic PK-59 tumors was analyzed by immunohistochemistry. The hypovascular region showed significantly higher expression of hypoxia-inducible factor (HIF)-1α and LDHA compared with the perivascular region (Figures 5A and 5B), These results suggest that hypoxia and nutritional starvation promote glycolysis in non-glycolytic PDAC tumors.

Next, we examined whether hypoxia and glucose deprivation promote glycolysis in non-glycolytic PDAC cells. Non-glycolytic PK-59 cells were exposed to hypoxia (1% O_2_) and low-glucose (90 mg/L) conditions in the hypoxia and low-glucose (HL) group or maintained under normoxic (21% O_2_) and normal glucose (2000 mg/L) conditions in the control normal oxygen and normal glucose (NN) group. The HL group showed significantly increased glucose uptake and lactate secretion compared with the control NN group (Figure 5C). The HL group further demonstrated increased expression of HIF-1α, GLUT1, and LDHA proteins and decreased expression of OGDH protein, compared with the control NN group (Figure 5D). By contrast, the expression of GDH1/2 and IDH1 proteins was similar between groups (Figure 5D). These results suggest that hypoxia and glucose deprivation promote glycolysis in non-glycolytic PDAC cells via LDHA activation and OGDH suppression.

To evaluate whether hypoxia and glucose deprivation promote virus sensitivity in non-glycolytic PDAC cells, non-glycolytic PK-59 cells in the HL and NN groups were infected with OBP-702, and cell viability was then analyzed using the XTT assay. OBP-702 significantly decreased the viability of PK-59 cells in the HL group more strongly than in the control NN group (Figure 5E). OBP-702 treatment (10 MOI) in the HL group resulted in increased expression of E1A, p53, and cleaved PARP proteins more strongly than in the control NN group (Figure 5F). OBP-702 treatment (100 MOI) in the HL group increased the expression of cleaved PARP protein more strongly than in the control NN group, although the expression of E1A and p53 proteins in the HL group was lower than in the control NN group. To investigate whether hypoxia and glucose deprivation enhance virus replication in non-glycolytic PDAC cells, non-glycolytic PK-59 cells in the HL and NN groups were infected with OBP-702, and the expression of E1A DNA and p53 mRNA was analyzed using real-time PCR (Figures 5G and 5H). OBP-702 significantly increased the expression of E1A DNA and p53 mRNA in the HL group more strongly than in the NN group. These results suggest that hypoxia and glucose deprivation promote virus sensitivity in non-glycolytic PDAC cells.

To investigate whether hypoxia and glucose deprivation increase the expression of adenovirus receptors in non-glycolytic PDAC cells, non-glycolytic PK-59 cells in the HL and NN groups were analyzed by FACS analysis. The expression of CAR and integrins αVβ3 and αVβ5 was not increased in non-glycolytic PK-59 cells in the HL group compared with the NN group (Figure S11). These results suggest that hypoxia and glucose deprivation increase virus sensitivity in non-glycolytic PDAC cells independent of adenovirus receptor modulation.

### Pharmacological activation of glycolysis improves virus sensitivity in non-glycolytic PDAC cells

**Figure 6:**
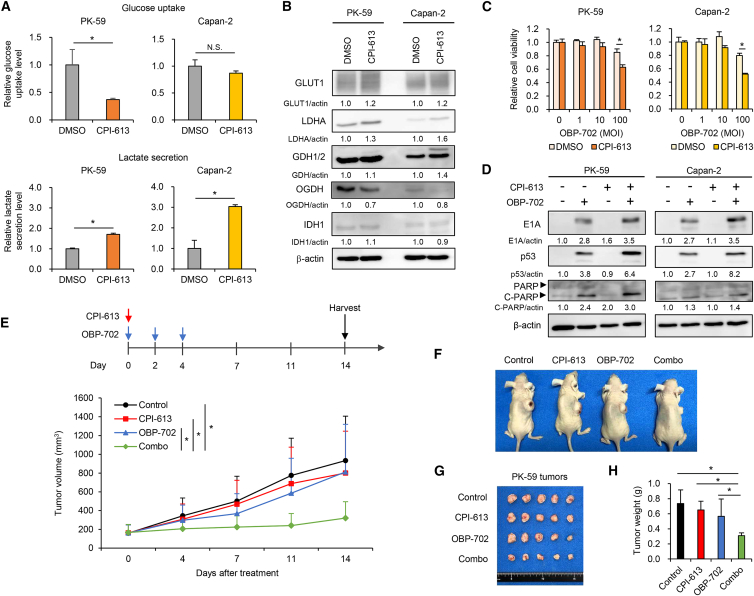
Combination treatment with CPI-613 enhances the antitumor efficacy of OBP-702 against non-glycolytic PK-59 tumors (A) The upper graphs show glucose uptake in non-glycolytic PDAC cells treated with CPI-613 (150 μM) or vehicle (DMSO). The lower graphs show lactate secretion by non-glycolytic PDAC cells treated with CPI-613 (150 μM) or DMSO. Data are expressed as mean (SD) of independent experiments (*n* = 3). The statistical significance of differences between two groups was determined using the Student’s *t* test. (B) Lysates of non-glycolytic PDAC cells treated with CPI-613 or DMSO were subjected to western blot analysis for GLUT1, LDHA, GDH1/2, OGDH, and IDH1. β-Actin was assayed as a loading control. (C) Cells were treated with CPI-613 (150 μM) or vehicle (DMSO) and then infected with OBP-702 at the indicated MOIs for 72 h. Cell viability was quantified using the XTT assay and calculated relative to the mock-infected group. Data are expressed as mean (SD) of independent experiments (*n* = 5). The statistical significance of differences between two groups was determined using the Student’s *t* test. (D) Cell lysates were subjected to western blot analysis for E1A, p53, PARP, and C-PARP. β-Actin was assayed as a loading control. The expression level of each protein was calculated relative to that of DMSO-treated or mock-treated cells, which was set at 1.0. (E) PK-59 tumor-bearing mice received intratumoral injections of OBP-702 (blue arrows) every other day for 3 cycles. CPI-613 (red arrows) was intraperitoneally injected into the peritoneal cavity of tumor-bearing mice. Data are expressed as mean (SD) of independent experiments (*n* = 5). The statistical significance of differences between four groups was determined using one-way ANOVA followed by Turkey’s multiple comparison procedure. (F) Representative photographs of tumor-bearing mice in each group. (G) Photographs of PK-59 tumors in each group. (H) Tumor weight of each group. Data are expressed as mean (SD) of independent experiments (*n* = 5). The statistical significance of differences between four groups was determined using one-way ANOVA followed by Turkey’s multiple comparison procedure. N.S., not significant; ∗, *p* < 0.05.

The mitochondrial metabolism inhibitor CPI-613 suppresses OGDH expression in PDAC cells.^35^ Therefore, we investigated the therapeutic potential of CPI-613 for glycolysis activation in non-glycolytic PDAC cells. Non-glycolytic PK-59 and Capan-2 cells were treated with CPI-613 or control vehicle (DMSO) and analyzed using an XTT assay, a metabolic assay, and western blotting. CPI-613 (>150 μM) significantly suppressed the viability of non-glycolytic PK-59 cells, whereas the viability of non-glycolytic Capan-2 was suppressed by CPI-613 (>200 μM) (Figure S12). PK-59 cells were more sensitive to CPI-613 than Capan-2 cells. CPI-613 (150 μM) significantly increased lactate secretion in non-glycolytic PDAC cells compared with control treatment, whereas glucose uptake was not affected or, conversely, decreased by CPI-613 treatment (Figure 6A). CPI-613 increased the expression of GLUT1 and LDHA and decreased the expression of OGDH compared with control treatment, whereas the expression of GDH1/2 and IDH1 did not differ between the groups (Figure 6B). These results suggest that CPI-613 activates glycolysis in non-glycolytic PDAC cells by upregulating LDHA expression and downregulating OGDH expression.

To evaluate whether CPI-613 promotes virus sensitivity, non-glycolytic PDAC cells pretreated with CPI-613 (150 μM) for 48 h were treated with OBP-702 for 72 h. Pretreatment with CPI-613 significantly promoted OBP-702-mediated cytopathic activity more strongly than in the control group (Figure 6C). The expression of p53 protein was not increased by pretreatment with CPI-613 (150 μM) alone in non-glycolytic PDAC cells (Figure 6D). By contrast, pretreatment with CPI-613 increased OBP-702-mediated upregulation of E1A, p53, and cleaved PARP protein expression more strongly than in the control group (Figure 6D), suggesting that CPI-613-mediated enhancement of virus sensitivity occurs in non-glycolytic PDAC cells.

To evaluate whether CPI-613 affects the expression of adenovirus receptors in non-glycolytic PDAC cells, non-glycolytic PK-59 and Capan-2 cells treated with CPI-613 (150 μM) for 48 h were analyzed by FACS analysis. CPI-613 treatment increased the expression of CAR and integrins αVβ3 and αVβ5 in PK-59 cells and, conversely, decreased it in Capan-2 cells (Figure S13). These results suggest that CPI-613 upregulates the expression of adenovirus receptors in non-glycolytic PK-59 cells.

To investigate whether CPI-613 enhances virus replication in non-glycolytic PDAC cells, non-glycolytic PK-59 and Capan-2 cells pretreated with CPI-613 were infected with OBP-702 (10 MOI) for 2 h. After replacement of the culture medium containing viruses with fresh medium, the expression of E1A DNA and p53 mRNA was analyzed at 2, 24, and 48 h after infection using real-time PCR (Figure S14). CPI-613 significantly increased the expression of E1A DNA and p53 mRNA in non-glycolytic PDAC cells. These results suggest that CPI-613 promotes virus replication in non-glycolytic PDAC cells.

To further evaluate whether CPI-613 promotes the *in vivo* antitumor efficacy of OBP-702, we examined a subcutaneous tumor model with non-glycolytic PK-59 cells. Monotherapy with CPI-613 or OBP-702 did not suppress the growth of PK-59 tumors (Figures 6E and 6F). However, combination therapy with CPI-613 and OBP-702 significantly suppressed the growth of PK-59 tumors compared with monotherapy or control treatment (Figures 6E and 6F). The tumor weight in the combination group was significantly decreased compared with that in the monotherapy or control group (Figures 6G and 6H). These results suggest that CPI-613 enhances the therapeutic efficacy of OBP-702 against non-glycolytic PDAC tumors.

### Metabolic parameters determined by PET/CT imaging are potent biomarkers of virus sensitivity in PDAC tumors

**Figure 7:**
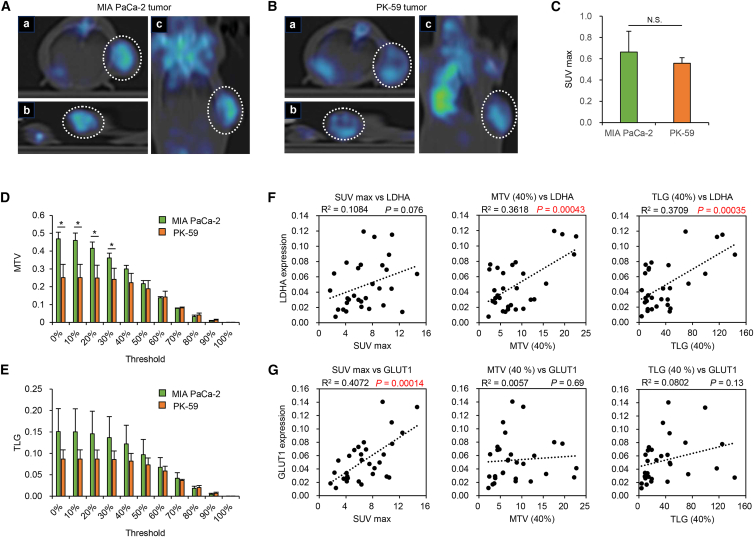
Investigation of the relationship between PET/CT metabolic parameters and glycolytic activity of PDAC tumors (A and B) PET/CT images of MIA PaCa-2 tumor (A) and PK-59 tumor (B). The upper left (a) shows the horizontal section, whereas the lower left (b) shows the sagittal section, and the right (c) shows the coronal section. Dotted circles indicate the tumor area. (C) Comparison of SUVmax values for MIA PaCa-2 and PK-59 tumors. Data are expressed as mean (SD) of independent experiments (*n* = 3). The statistical significance of differences between two groups was determined using the Student’s *t* test. (D and E) Comparison of MTV (D) and TLG (E) values for MIA PaCa-2 and PK-59 tumors at the indicated thresholds. Data are expressed as mean (SD) of independent experiments (*n* = 3). The statistical significance of differences between two groups was determined using the Student’s *t* test. (F and G) Scatter diagrams demonstrating correlations between expression of LDHA (F) or GLUT1 (G) and preoperative SUVmax (left), MTV (40%) (center), and TLG (40%) (right) values in patients with PDAC (*n* = 30). The statistical significance of the correlations in the scatterplots was determined using Pearson’s correlation analysis. N.S., not significant; ∗, *p* < 0.05.

PET/CT imaging is a diagnostic tool used to assess glucose-related metabolic activity within tumors in the clinical setting. Several parameters are useful in this regard for assessing the malignant potential of advanced PDAC tumors.^36^^,^^37^ Maximum standardized uptake value (SUVmax) is defined as the highest SUV of 18F-fluorodeoxyglucose (FDG) in the tumor. Metabolic tumor volume (MTV) is defined as the estimated volume of the tumor exhibiting increased FDG uptake. Total lesion glycolysis (TLG) is defined as the product of the average SUV (SUVmean) of the total tumor multiplied by MTV. Therefore, to evaluate whether metabolic parameters determined using PET/CT imaging could be used to distinguish glycolytic and non-glycolytic PDAC tumors, we obtained subcutaneous tumors with glycolytic MIA PaCa-2 and non-glycolytic PK-59 cells at similar tumor sizes (approximately 10 mm in diameter) and analyzed the metabolic parameters using PET/CT imaging (Figures 7A and 7B). There were no significant differences in the SUVmax values between the groups (Figure 7C). When both tumor types were compared at a threshold of <30% SUVmax, the MTV of glycolytic MIA PaCa-2 tumors was significantly higher than that of non-glycolytic PK-59 tumors (Figure 7D). By contrast, there were no significant differences in TLG between tumor types at any SUVmax threshold (Figure 7E). These results suggest that MTV is a potent biomarker associated with the determination of glycolytic activity in PDAC tumors.

To further evaluate whether metabolic parameters determined by PET/CT imaging are associated with glycolytic activity in clinical PDAC tumors, we analyzed the relationship between the expression of tumoral glycolytic markers and PET/CT imaging parameters in 30 patients with PDAC. The expression of LDHA was significantly correlated with MTV and TLG when calculated at a threshold of 40% SUVmax (Figure 7F). Expression of GLUT1 was significantly correlated with SUVmax, but MTV and TLG were not (Figure 7G). By contrast, there was no significant correlation between the expression of HIF-1α and metabolic parameters (Figure S15). These results suggest that MTV is a useful biomarker for predicting glycolytic activity with LDHA expression in PDAC tumors.

## Discussion

Glycolytic PDAC cells are associated with resistance to antitumor therapies, including chemotherapy.^38^ Therefore, novel therapeutic strategies to eliminate glycolytic PDAC cells are urgently needed to improve the clinical outcomes of patients with PDAC. In this study, we demonstrated that the metabolic subtypes of PDAC cells differ in sensitivity to oncolytic adenoviruses. Glycolytic PDAC cells were more sensitive to OBP-301 and OBP-702 compared with non-glycolytic PDAC cells via glycolysis activation. OBP-702 was superior to OBP-301 in terms of virus sensitivity in PDAC cells via p53-mediated modulation of glutamine metabolism. The virus sensitivity of non-glycolytic PDAC cells was enhanced through glycolysis activation induced by exposure to HL conditions or treatment with the mitochondrial metabolic inhibitor CPI-613. Moreover, metabolic parameters determined by PET/CT imaging were useful as a tool to distinguish glycolytic and non-glycolytic PDAC tumors. Thus, glycolytic PDAC cells may be a potent therapeutic target for treatment with oncolytic adenoviruses as precision medicine, and combination treatment with CPI-613 may improve the therapeutic potential of oncolytic adenoviruses against virotherapy-resistant non-glycolytic PDAC tumors.

Glycolytic PDAC cells with high LDHA expression were more sensitive to oncolytic adenoviruses compared with non-glycolytic cells with low LDHA expression in both *in vitro* and *in vivo* settings (Figures 1 and 4). As the ERK inhibitor SCH772984 suppressed the therapeutic potential of oncolytic adenoviruses in glycolytic PDAC cells by downregulating the expression of LDHA (Figure 2), the ERK-LDHA signaling pathway may play a crucial role in maintaining the virus sensitivity of PDAC cells. With regard to the underlying mechanism of glycolytic activation in PDAC cells, KRAS activation has been shown to induce glycolysis in these cells.^39^ The ERK and c-Jun N-terminal kinase signaling pathways, which are downstream of KRAS, have been shown to regulate glycolysis in cancer cells.^40^ Laevskaya et al. suggested that adenovirus-mediated lactate accumulation is associated with autophagy-related cell death.^41^ As we demonstrated that OBP-301 and OBP-702 induce autophagy-related cell death in human PDAC cells,^20^^,^^42^ glycolytic PDAC cells may be potent therapeutic targets for oncolytic adenoviruses.

Glutamine metabolism is an important factor in promoting adenovirus replication^24^ in addition to glucose metabolism. Viral p53 activation promoted glutamine metabolism, which in turn promoted the biosynthesis of α-KG by downregulating OGDH expression (Figure 3). However, whether OBP-702-mediated p53 activation promotes viral genome synthesis via α-KG accumulation remains unclear. α-KG has been shown to play a crucial role in supporting adenovirus replication in cancer cells.^21^ With regard to the role of glutamine metabolism in PDAC, several reports have focused on glutamine transfer and biogenesis pathways within PDAC,^43^^,^^44^^,^^45^ in which p53 activation contributes to glutamine biogenesis during glutamine depletion.^46^^,^^47^ To investigate the role of p53-mediated α-KG accumulation in viral genome synthesis, further experiments are warranted to evaluate whether p53-armed OBP-702 has therapeutic potential to increase viral genome synthesis in PDAC cells lacking α-KG accumulation by genetically or pharmacologically suppressing glutaminolysis-related enzymes.

PDAC cells are frequently exposed to hypoxia and low-nutrient conditions in the TME due to poor vascularization.^12^ Non-glycolytic PDAC tumors showed a significant relationship between HIF-1α and LDHA expression (Figure 5). Hypoxia and glucose deprivation induced glycolysis in non-glycolytic PDAC cells (Figure 5). These findings suggest that glycolytic activity is affected by TME factors. PDAC tumors reportedly exhibit metabolic heterogeneity.^48^ Accumulating evidence suggests that cancer-associated fibroblasts play a crucial role in modulating metabolism in PDAC tumors with a dense stroma.^49^ Moreover, Sharick et al. demonstrated that PDAC patient-derived organoids mimic this metabolic heterogeneity and resistance to chemotherapy.^50^ Therefore, it is necessary to evaluate whether OBP-702 has therapeutic potential against PDAC xenograft tumor models with stromal tissues or PDAC organoid models exhibiting metabolic heterogeneity.

Combination treatment with CPI-613 improved the therapeutic efficacy of OBP-702 against non-glycolytic PDAC cells by promoting glycolysis (Figure 6), suggesting that CPI-613-mediated metabolic modification could be useful for increasing the therapeutic potential of oncolytic adenoviruses. With regard to the underlying mechanism of CPI-613-mediated metabolic modulation, CPI-613 inhibits the expression of OGDH and pyruvate dehydrogenase (the enzyme that catalyzes the conversion of pyruvate to acetyl-CoA) in the TCA cycle.^51^ Recently, Gao et al. demonstrated that CPI-613 suppresses lipid metabolism in PDAC cells, leading to the enhancement of apoptosis and autophagy.^52^ As OBP-702 induces apoptosis- and autophagy-related PDAC cell death,^20^^,^^42^ further investigation is needed to explore the underlying mechanism of the synergistic effect of oncolytic adenoviruses and CPI-613 in combination for clinical applications. To evaluate the underlying mechanism of CPI-613-mediated modulation of viral replication efficiency and adenovirus receptors, further experiments involving transcriptomic, metabolomic, and proteomic analyses would help determine how CPI-613 influences viral replication efficiency and virus infectivity at the molecular level.

The metabolic heterogeneity in tumors has been shown to be associated with tumor progression and chemoresistance in a variety of malignancies.^53^^,^^54^ Therefore, it is necessary to develop therapeutic strategies that target metabolic heterogeneity in tumors. Udumula et al. demonstrated that CPI-613 suppressed mitochondrial metabolism, resulting in enhanced chemosensitivity and suppression of tumor growth in chemoresistant ovarian cancer xenograft models.^55^ Arnold et al. showed that CPI-613 synergistically enhanced the antitumor effect of chemotherapy, and combination therapy with CPI-613 and chemotherapy suppressed tumor growth in colorectal cancer xenograft models.^56^ Thus, combination with CPI-613 may be applicable to promote the therapeutic potential of antitumor modalities, including chemotherapy and oncolytic virotherapy, against ovarian and colorectal cancers with metabolic heterogeneity. For clinical application of combination therapy with OBP-702 and CPI-613, further *in vivo* experiments using CPI-613 over a prolonged period are warranted to evaluate the safety and therapeutic impact of combination therapy on the survival of mice bearing non-glycolytic PDAC tumors and other cancers.

Assessing glycolytic activity in PDAC tumors enables clinicians to predict responsiveness to antitumor therapies, as glycolytic PDAC cells are resistant to chemotherapy.^38^ *In vivo* experiments in the present study demonstrated that MTV is associated with glycolytic activity in PDAC tumors (Figure 7C). Moreover, the expression of LDHA in clinical PDAC tissues was significantly associated with MTV and TLG parameters (Figure 7D). These findings suggest that MTV and TLG values are useful biomarkers for predicting glycolytic activity in PDAC tumors. The MTV value has been associated with poor prognosis in patients with advanced PDAC.^36^^,^^37^ The TLG value is reportedly correlated with local progression in patients with advanced PDAC.^37^ Thus, PET/CT imaging may be a useful approach for assessing glycolytic activity to predict not only malignancy potential but also the therapeutic potential of oncolytic virotherapy in patients with PDAC. As we are now preparing to conduct a phase I clinical study of monotherapy with OBP-702 in patients with advanced PDAC, it will be possible to assess the relationship between treatment response to OBP-702 and PET/CT-based metabolic parameters in oncolytic virotherapy with OBP-702.

PDAC is known to be an immune-evasive disease with poor immune response and resistance to immune checkpoint inhibitors.^57^ We previously demonstrated that OBP-702 induces immunogenic cell death in human and murine PDAC cells.^42^ Recently, Yang et al. demonstrated that suppression of mitochondrial metabolism by CPI-613 induced endoplasmic reticulum stress, resulting in enhanced antitumor immune responses with tumor infiltration of cytotoxic T cells in a syngeneic mouse model of orthotopic head and neck cancer.^58^ However, whether combination therapy with CPI-613 and OBP-702 affects antitumor immunity in murine PDAC tumors remains unclear. Therefore, it is necessary to evaluate the immunomodulatory effect of combination therapy with OBP-702 and CPI-613 using murine PDAC tumor models.

In conclusion, we demonstrated that non-glycolytic PDAC cells are less sensitive to oncolytic adenoviruses than glycolytic cells. TME-associated factors, including hypoxia and glucose deprivation, as well as treatment with the mitochondrial metabolism inhibitor CPI-613, were shown to modulate the metabolic reprogramming of non-glycolytic cells into glycolytic cells, leading to enhanced therapeutic potential of oncolytic adenoviruses. Thus, combination therapy with OBP-702 and CPI-613 may be a promising antitumor strategy to overcome low virus sensitivity in PDAC tumor cells. Moreover, metabolic parameters determined using PET/CT imaging may serve as useful predictive biomarkers for assessing virus sensitivity in patients with PDAC.

## Materials and methods

### Cell lines

Two human PDAC cell lines (MIA PaCa-2 and Capan-2) were obtained from the American Type Culture Collection (Manassas, VA, USA). Two additional human PDAC cell lines (PK-45H, PK-59) were obtained from the Cell Resource Center for Biomedical Research, Institute of Development, Aging and Cancer, Tohoku University (Sendai, Japan). Cells were cultured for no longer than 5 months following resuscitation. Authentication was not performed by the authors. MIA PaCa-2 cells were maintained in Dulbecco’s Modified Eagle’s medium supplemented with 10% fetal bovine serum (FBS). Capan-2 cells were maintained in McCoy’s 5A medium supplemented with 10% FBS. PK-45H and PK-59 cells were maintained in RPMI 1640 medium supplemented with 10% FBS. All media were supplemented with 100 U/mL penicillin and 100 μg/mL streptomycin. Cells were routinely maintained at 37°C in a humidified atmosphere with 5% CO_2_.

To maintain PDAC cells under hypoxic conditions, cells were incubated for 72 h in a hypoxic chamber (Modular Incubator Chamber; Billups-Rothenberg, Del Mar, CA, USA) filled with a gas mixture of 1% O_2_, 5% CO_2_, and 94% N_2_. To maintain PDAC cells under glucose-deprived conditions, glucose-free medium supplemented with 10% FBS and an appropriate amount of glucose (final concentration: 90 mg/L) was used.

### Recombinant adenoviruses

The telomerase-specific replication-competent adenovirus OBP-301 (suratadenoturev), in which the promoter element of the *hTERT* gene drives the expression of *E1A* and *E1B* genes (Figure S1A), was constructed and characterized previously.^15^ OBP-702 was constructed by modifying OBP-301 to express the exogenous *p53* gene, by inserting a human wild-type *p53* gene expression cassette driven by the Egr-1 promoter into the E3 region of OBP-301 (Figure S1A).^18^ OBP-401 (TelomeScan) was constructed by modifying OBP-301 to express GFP by inserting a *GFP* gene expression cassette into the E3 region of OBP-301 (Figure S1A).^30^ Ad-p53 is a replication-defective adenovirus serotype 5 (Ad5) vector with a human wild-type *p53* gene expression cassette in the E1 region (Figure S1B). DL312 is an E1A-deleted Ad5 vector (Figure S1B). Recombinant adenoviruses were purified using cesium chloride step gradients, and virus titers were determined using a plaque-forming assay with 293 cells. Viruses were stored at −80°C.

### Reagents

ERK1/2 inhibitor SCH772984 was obtained from CHEMIETEK (Indianapolis, IN, USA). 2DG was purchased from Sigma-Aldrich (St. Louis, MO, USA). CPI-613 (devimistat) was obtained from Cayman Chemical (Ann Arbor, MI, USA).

### Metabolic assays

Cells were seeded in a 96-well plate at 5 × 10^3^ cells/well 24 h before treatment. The cells were then infected with OBP-301, OBP-702, Ad-p53, or DL312 or treated with SCH772984, 2DG, or CPI-613 at the indicated doses for 24 h. Glucose uptake, lactate secretion, glutamine consumption, and intracellular α-KG levels were measured using the Glucose Uptake-Glo Assay kit (Promega), Lactate-Glo assay kit (Promega), Glutamine-Glutamate-Glo-Assay Kit (Promega), and α-KG Assay Kit (Sigma-Aldrich), respectively, according to the manufacturers’ protocols.

### Western blot analysis

Cells were seeded in a 100-mm dish at a density of 1 × 10^5^ cells/dish 24 h before treatment. For monotherapy, cells were infected with adenoviruses or treated with reagents at the indicated doses for 48 h. For combination therapy, cells pretreated with CPI-613 for 48 h were infected with OBP-702 for 72 h. Whole-cell lysates were prepared in lysis buffer (50 mM Tris-HCl [pH 7.4], 150 mM NaCl, 1% Triton X-100) containing a protease inhibitor cocktail (Complete Mini; Roche, Indianapolis, IN, USA). Proteins were electrophoresed on 8%–10% sodium dodecyl sulfate polyacrylamide gels and transferred to polyvinylidene difluoride membranes (Hybond-P; GE HealthCare, Buckinghamshire, UK). Membranes were blocked with Blocking-One (Nacalai Tesque, Kyoto, Japan) at room temperature for 30 min. The primary antibodies used were: rabbit GLUT1 monoclonal antibody (mAb) (ab115730; Abcam, Cambridge, UK), rabbit anti-LDHA mAb (3582; Cell Signaling Technology, Beverly, MA, USA), mouse anti-Ad5 E1A mAb (554155; BD Bioscience, Franklin Lakes, NJ, USA), mouse anti-p53 mAb (18032; Cell Signaling Technology), rabbit PARP polyclonal antibody (pAb) (9542; Cell Signaling Technology), rabbit anti-p44/42 (ERK1/2) mAb (4695; Cell Signaling Technology), rabbit GDH1/2 mAb (12793; Cell Signaling Technology), rabbit-anti-OGDH mAb (26865; Cell Signaling Technology), rabbit anti-IDH1 pAb (12332-1-AP; Proteintech, Rosemont, IL, USA), rabbit anti-HIF-1α mAb (14179; Cell Signaling Technology), and mouse anti–β-actin mAb (A5441; Sigma-Aldrich, St. Louis, MO, USA). Secondary antibodies used were: horseradish peroxidase–conjugated antibodies against mouse IgG (NA931; GE Healthcare; Chicago, Illinois, USA) or rabbit IgG (NA934; GE Healthcare). Immunoreactive bands on the blots were visualized using enhanced chemiluminescence substrates (ECL Plus; GE Healthcare).

### GFP expression assay

Cells were seeded in 24-well plates at a density of 1 × 10^4^ cells/well 24 h before treatment. For monotherapy, cells were infected with OBP-401 at MOIs of 100 PFU/cell. For combination therapy, OBP-401 and each reagent were administered concurrently at the indicated doses. Twenty-four, 48, or 72 h after treatment, cells were fixed with 4% paraformaldehyde and permeabilized with methanol. 4,6-diamidino-2-phenylindole (DAPI) was used to identify nuclei. Three randomly selected fields per well were photographed using a confocal laser scanning biological microscope (IX83; Olympus; Tokyo, Japan), and GFP inensity was calculated using ImageJ software.

### Cell viability assay

Cells were seeded in 96-well plates at a density of 10^3^ cells/well 24 h before treatment. For monotherapy, cells were infected with OBP-301, OBP-702, or treated with SCH772984, 2DG, or CPI-613 at the indicated doses for 24 and 72 h. For combination therapy, cells pretreated with CPI-613 for 48 h were infected with OBP-702 for 72 h. Cell viability was determined using a Cell Proliferation Kit II (Roche, Indianapolis, IN, USA) according to the manufacturer’s protocol.

### *In vivo* subcutaneous xenograft tumor model

Animal experimental protocols were approved by the Ethics Review Committee for Animal Experimentation of Okayama University School of Medicine (No. OKU-2021180). To develop subcutaneous PDAC tumors with different metabolic phenotypes, MIA PaCa-2 cells (5 × 10^6^ cells/mouse) or PK-59 cells (2 × 10^6^ cells/mouse) were inoculated into the right flank of female BALB/c-nu/nu mice (CLEA Japan, Tokyo, Japan). For monotherapy, when tumors had reached a diameter of 7–8 mm, OBP-702 (5 × 10^7^ PFU/50 μL) or PBS was injected intratumorally on days 0, 2, and 4. For combination treatment with CPI-613, CPI-613 (12.5 mg/kg body weight) or corn oil was injected intraperitoneally on day 0. The perpendicular diameters of each tumor were measured every 3–4 days, and tumor volume was calculated using the following formula: tumor volume (mm^3^) = *a* × *b*^2^ × 0.5, where *a* represents the longest diameter, *b* represents the shortest diameter, and 0.5 is the constant used to estimate the volume of an ellipsoid.

### Immunohistochemistry

Subcutaneous tumors were fixed in 10% neutralized formalin and embedded in paraffin blocks. Tissue sections (4 μm) were deparaffinized in xylene and rehydrated through a graded ethanol series. Endogenous peroxidases were blocked by incubation with 3% H_2_O_2_ for 10 min, and antigen retrieval was performed by boiling the samples in citrate buffer or EDTA buffer for 14 min in a microwave oven. Samples were incubated with primary antibodies overnight at 4°C, followed by incubation with peroxidase-linked secondary antibodies for 30 min at room temperature. For sections from *in vivo* subcutaneous tumors using anti-mouse antibodies, the Histofine Mouse Stain Kit (Nichirei Bioscience, Tokyo, Japan) was used. Primary antibodies included rabbit anti-LDHA mAb (3582; Cell Signaling Technology), rabbit anti-GLUT1 mAb (ab115730; Abcam), rabbit anti-IDH1 pAb (12332-1-AP; Proteintech), and mouse anti–HIF-1α mAb (NB100-296; Novus Biologicals, Colorado, USA). After 3,3-diaminobenzidine (DAB) staining for signal generation and counterstaining with Mayer’s hematoxylin, samples were dehydrated and mounted onto coverslips. DAB staining intensity per number of nuclei was calculated from five different randomly selected fields using ImageJ Fiji software, following the protocol reported by Crowe et al.^59^

### Quantitative real-time RT-PCR analysis

To evaluate the copy number of E1A DNA in virus-infected PDAC cells, cells were seeded in 6-well plates at a density of 2 × 10^5^ cells/well 24 h before infection and infected with OBP-301 or OBP-702 at an MOI of 10 PFU/cell. After infection for 2 h, the culture medium containing viruses was removed and replaced with fresh medium. Total DNA was extracted from cells at 2, 24, and 48 h after infection using a QIAmp DNA Mini kit (Qiagen, Hilden, Germany). *E1A* copy number was determined by quantitative real-time PCR using the StepOnePlusTM real-time PCR system (Applied Biosystems, Carlsbad, CA, USA) and TaqMan gene expression assays (Applied Biosystems). The sequences of the primers and probe used in this experiment were: E1A primers, 5′-CCT GAG ACG CCC GAC ATC-3′ and 5′-GGA CCG GAG TCA CAG CTA TCC-3’; E1A probe, 5′-FAM-CTG TGT CTA GAG AAT GC-MGB-3’. Data were analyzed using StepOne Software (Applied Biosystems).

To evaluate the expression of hTERT mRNA in PDAC cells, cells were seeded in 6-well plates at a density of 2 × 10^5^ cells/well, and after 24 h, total RNA was extracted from the cells using a miRNeasy Mini Kit (Qiagen, Valencia, CA, USA). To evaluate the expression of p53 mRNA in virus-infected PDAC cells, cells were seeded in 6-well plates at a density of 2 × 10^5^ cells/well 24 h before infection. After 2 h of infection, the culture medium containing viruses was removed and replaced with fresh medium. Total RNA was extracted from cells at 2, 24, and 48 h after infection with OBP-301 or OBP-702 at an MOI of 10 PFU/cell using the miRNeasy Mini Kit (Qiagen). cDNA was synthesized from 10 ng of total RNA using a TaqMan Reverse Transcription Kit (Applied Biosystems). The expression levels of hTERT, p53, and glyceride-hyde-3-phosphate dehydrogenase (GAPDH) mRNA were determined using quantitative real-time PCR on an Applied Biosystems StepOnePlus real-time PCR system (Applied Biosystems). Relative expression levels of hTERT mRNA or p53 mRNA were calculated using the 2^−ΔΔCt^ method after normalization to GAPDH mRNA.

### Patients with PDAC

This study was reviewed and approved by the Institutional Review Board of Okayama University (No. 2107-004). Thirty patients with PDAC who received preoperative FDG PET/CT (Siemens Biograph 16, Siemens Medical Solutions, Erlangen, Germany) at the Okayama Imaging Diagnostic Center and pancreatectomy without preoperative treatment at Okayama University Hospital between 2007 and 2017 were included in this study. Informed consent was obtained by the opt-out method. The sections containing the largest circumferential plane of the tumor was selected from the pancreatectomy specimen of each case, and immunostaining of tumor sections was performed to assess the metabolic parameters.

### Calculation of PET/CT metabolic parameters in patients with PDAC

Thirty patients with PDAC received preoperative 18F-FDG PET/CT imaging. Administration of 18F-FDG was performed intravenously with a body weight-adapted dose of 3.7 MBq/kg. PET/CT image acquisition started 60 min after 18F-FDG administration. PET/CT image analysis was performed using a syngo.via (Siemens Healthineers, Erlangen, Germany). For each tumor, SUVmax was determined on PET images. Prior to this, tumor margins were identified on diagnostic CT images and fused PET/CT images, and a round volume of interest (VOI) that included the entire lesion in the axial, sagittal, and coronal planes was placed in the PET dataset. MTV and mean SUV (SUVmean) were also calculated using an SUVmax threshold of 40%. TLG was calculated as (SUVmean) × (MTV).

### Calculation of PET/CT metabolic parameters using an *in vivo* subcutaneous xenograft tumor model

Animal experimental protocols were approved by the Ethics Review Committee for Animal Experimentation of Okayama University School of Medicine (no. OKU-2021833). To develop subcutaneous PDAC tumors with different metabolic phenotypes, glycolytic MIA PaCa-2 cells (5 × 10^6^ cells/mouse) or non-glycolytic PK-59 cells (2 × 10^6^ cells/mouse) were inoculated into the right flank of female BALB/c-nu/nu mice. 18F-FDG was injected into the tail vein of mice, and they were anesthetized by inhalation of 1.5%–2.0% isoflurane. 60 min after injection, PET scans were performed using a small-animal PET scanner (Clairvivo PET, Shimadzu, Kyoto, Japan), and the images were reconstructed using the 3D-DRAMA method. After the PET scan, CT data were obtained using a PET/CT system (Eminence Stargate; Shimadzu). PET and CT images were converted to DICOM format and fused using PMOD software version 3.3 (PMOD Technologies Ltd.). For each tumor, SUVmax was determined on PET images. A round VOI was drawn on PET/CT images to determine SUVmean, MTV, and TLG in tumors. SUV was calculated as follows: SUV = [tissue radioactivity in Bq/g tissue]/[injected radioactivity in Bq/g body weight].

### Statistical analysis

Data are expressed as the mean ± SD. For comparison between 2 groups, significant differences were assessed using the Student’s *t* test. For comparison of more than 2 groups, statistical significances were determined using one-way ANOVA followed by Turkey’s multiple comparison procedure. To examine correlations in the scatterplots, Pearson’s product-rate correlation coefficient was calculated. Statistical significance was defined as a *p* value less than 0.05.

## Data availability

All data generated or analyzed during this study are included in the main text or the supplemental information.

## Acknowledgments

We thank Takanori Sasaki for supporting the PET/CT imaging and Tomoko Sueishi, Yuko Hoshijima, and Tae Yamanishi for their excellent technical support. This study was supported in part by grants from the 10.13039/100009619Japan Agency for Medical Research and Development (17ck0106285h0001 and 20ck0106569h0001 to T.F.) and 10.13039/501100001691Japan Society for the Promotion of Science KAKENHI grants (JP16K10596 and JP21K07219 to H.T. and JP19H03731 and JP22H03148 to T.F.).

## Author contributions

Conception and design, H.T., S.Ka., and T.F.; development of methodology, R.S., H.T., and S.Ku.; acquisition of data, R.S., T.N., Y.K., M.Y., Y.N., H.I., and N.H.; analysis and interpretation of data, R.S., T.N., Y.K., M.Y., Y.N., H.I., and N.H.; writing, review, and/or revision of the manuscript, R.S., H.T., and T.F.; administrative, technical, or material support, Y.Ur.; study supervision, H.T., S.Ku., S.Ki., R.Y., Y.Um., S.Ka, and T.F.

## Declaration of interests

Y.Ur. is President and CEO of Oncolys BioPharma, Inc. H.T. and T.F. are consultants for Oncolys BioPharmathe , Inc.

## Supplemental information

Document S1. Figures S1–S15

Document S2. Article plus supplemental information
