# Supplementary material for: Mitochondrial inhibition enhances the sensitivity of pancreatic ductal adenocarcinoma cells to oncolytic adenovirus
Source: Mol Ther Oncol. 2026 Mar 18;34(2):201180. doi: 10.1016/j.omton.2026.201180 (PMC13084250; doi:10.1016/j.omton.2026.201180)
Supplement: Document S1. Figures S1–S15 [file mmc1.pdf]

OMTON, Volume *34*

Supplemental information

Mitochondrial inhibition enhances the sensitivity of pancreatic ductal adenocarcinoma cells to oncolytic adenovirus

Ryohei Shoji, Hiroshi Tazawa, Shinji Kuroda, Takeyoshi Nishiyama, Yoshinori Kajiwara, Motohiko Yamada, Yasuo Nagai, Hiroaki Inoue, Naoyuki Hashimoto, Satoru Kikuchi, Ryuichi Yoshida, Yuzo Umeda, Yasuo Urata, Shunsuke Kagawa, and Toshiyoshi Fujiwara

Supplemental Information

Materials and Methods

Flow cytometry

To analyze the expression of CAR and integrin αvβ3 and αvβ5, cells were incubated with mouse anti-CAR monoclonal antibody (mAb) (RmcB; Upstate, Lake Placid, NY, USA), rabbit anti-integrin αvβ3 polyclonal antibody (pAb) (bs-1310R; Bioss Antibodies, Woburn, MA, USA), and rabbit anti-integrin αvβ5 pAb (bs-1356R; Bioss Antibodies) or isotype control IgG for 60 min on ice. The cells were then labeled with fluorescent isothiocyanate (FITC)-conjugated rabbit anti-mouse IgG secondary antibody (A16161; Invitrogen, Carlsbad, CA, USA) or Alexa Fluor 647-conjugated goat anti-rabbit IgG secondary antibody (A-21245; Invitrogen) for 30 min and then analyzed using FACS Lyric (BD Biosciences). The mean fluorescence intensity (MFI) was determined by calculating the difference between the MFI of antibody-incubated and isotype control IgG-incubated cells.

GFP expression assay

To analyze the role of p53 activation in virus sensitivity, cells were seeded in 24-well plates at a density of 1×10^4 cells/well 24 h before infection with Ad-p53 or DL312 at an MOI of 100 PFU/cell, followed by infection with OBP-401 at an MOI of 100 PFU/cell. At 24 and 48 h after treatment, cells were fixed with 4% paraformaldehyde and permeabilized with methanol. DAPI was used to identify nuclei. Three randomly selected fields of each well were photographed using a confocal laser scanning biological microscope (IX83; Olympus, Tokyo, Japan), and the intensity of GFP was calculated using ImageJ software.

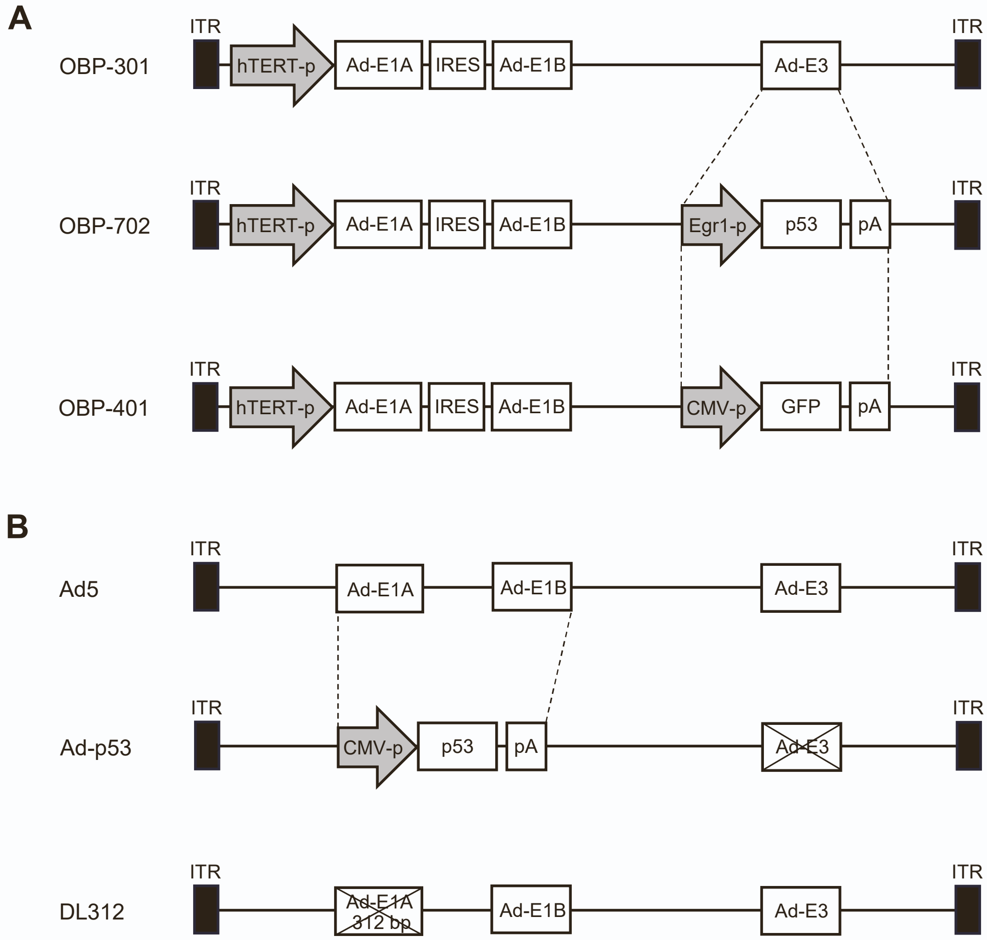

Figure S1. Schematic diagrams of structures of the oncolytic adenoviruses and replication-deficient adenoviruses. A, OBP-301 is a telomerase-specific, replication-competent oncolytic adenovirus, in which the hTERT promoter drives expression of the adenoviral E1A and E1B genes, which are linked by an IRES. OBP-702 is a p53-armed OBP-301 variant generated by inserting the Egr1 promoter-driven human wild-type p53 gene expression cassette into the E3 region of OBP-301. OBP-401 is a GFP-expressing OBP-301variant generated by inserting the CMV promoter-driven GFP gene expression cassette into the E3 region of OBP-301. **B**, Ad-p53 is a replication-defective adenovirus serotype 5 vector with a human wild-type p53 gene expression cassette at the E1 region and E3 region of Ad-p53 is deleted. DL312 is an E1A (312 bp)-deleted Ad5 vector.

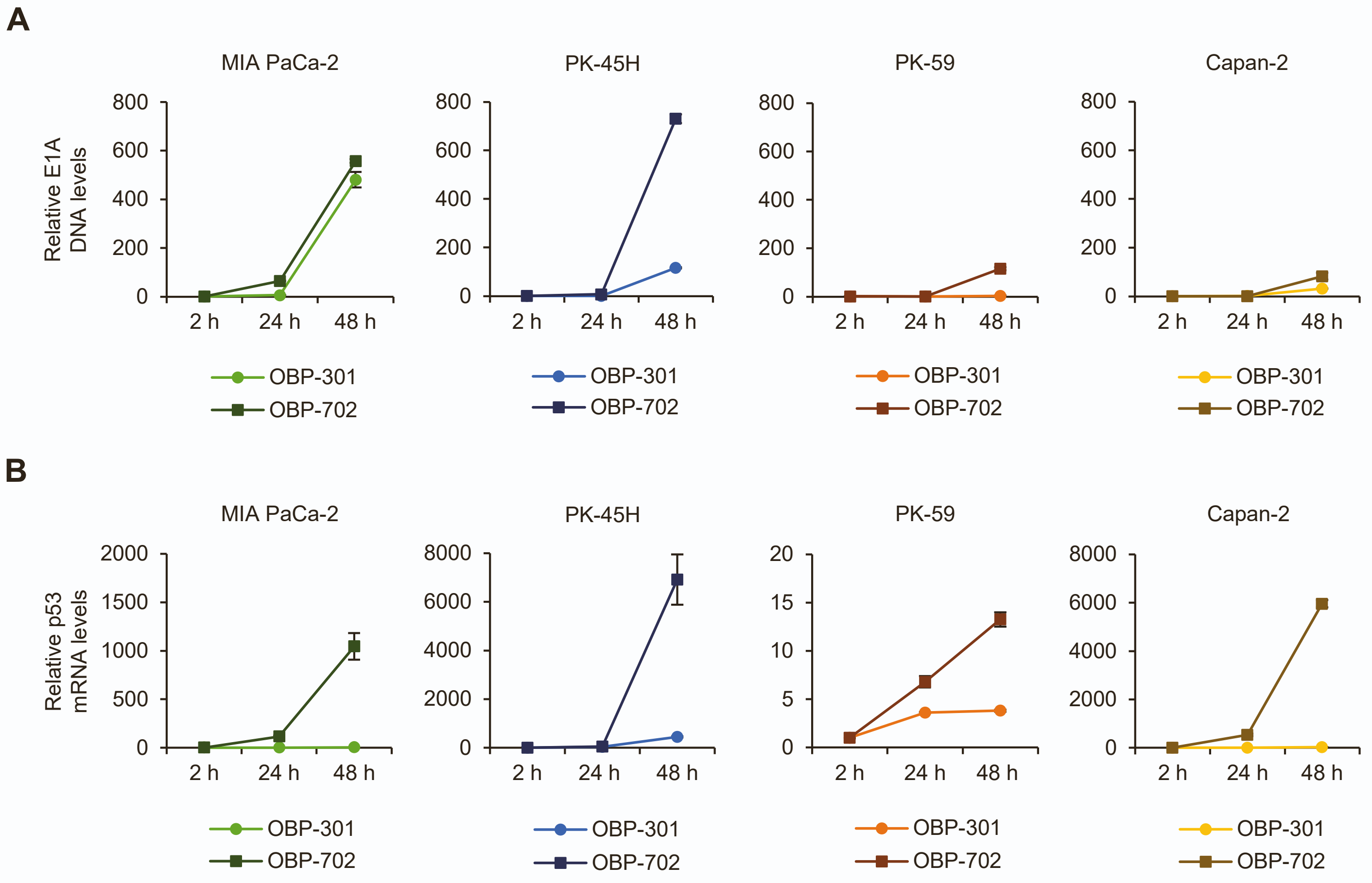

Figure S2. Change in the expression level of adenoviral E1A DNA and p53 mRNA over time in human PDAC cells infected with OBP-301 or OBP-702. A, B, Copy number of adenoviral E1A DNA (**A**) and expression of p53 mRNA (**B**) were analyzed by real-time RT-PCR. The relative copy number of E1A DNA and relative expression of p53 mRNA are presented as fold-increase compared with 2 h, which was set as 1.0. Data are expressed as mean value (SD) of independent experiment (n = 3).

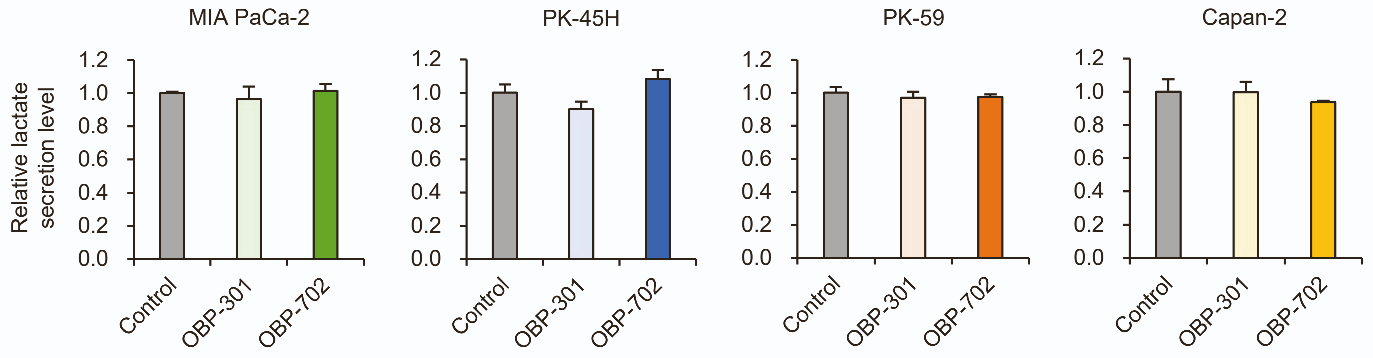

Figure S3. No change in lactate secretion by human PDAC cells after infection with OBP-301 or OBP-702. The amount of lactate secreted by virus-treated PDAC cells was analyzed using a metabolic assay with conditioned medium. The relative amount of lactate is presented as fold-increase compared with the mock-treated control, which was set as 1.0. Data are expressed as mean value (SD) of independent experiment (n = 3).

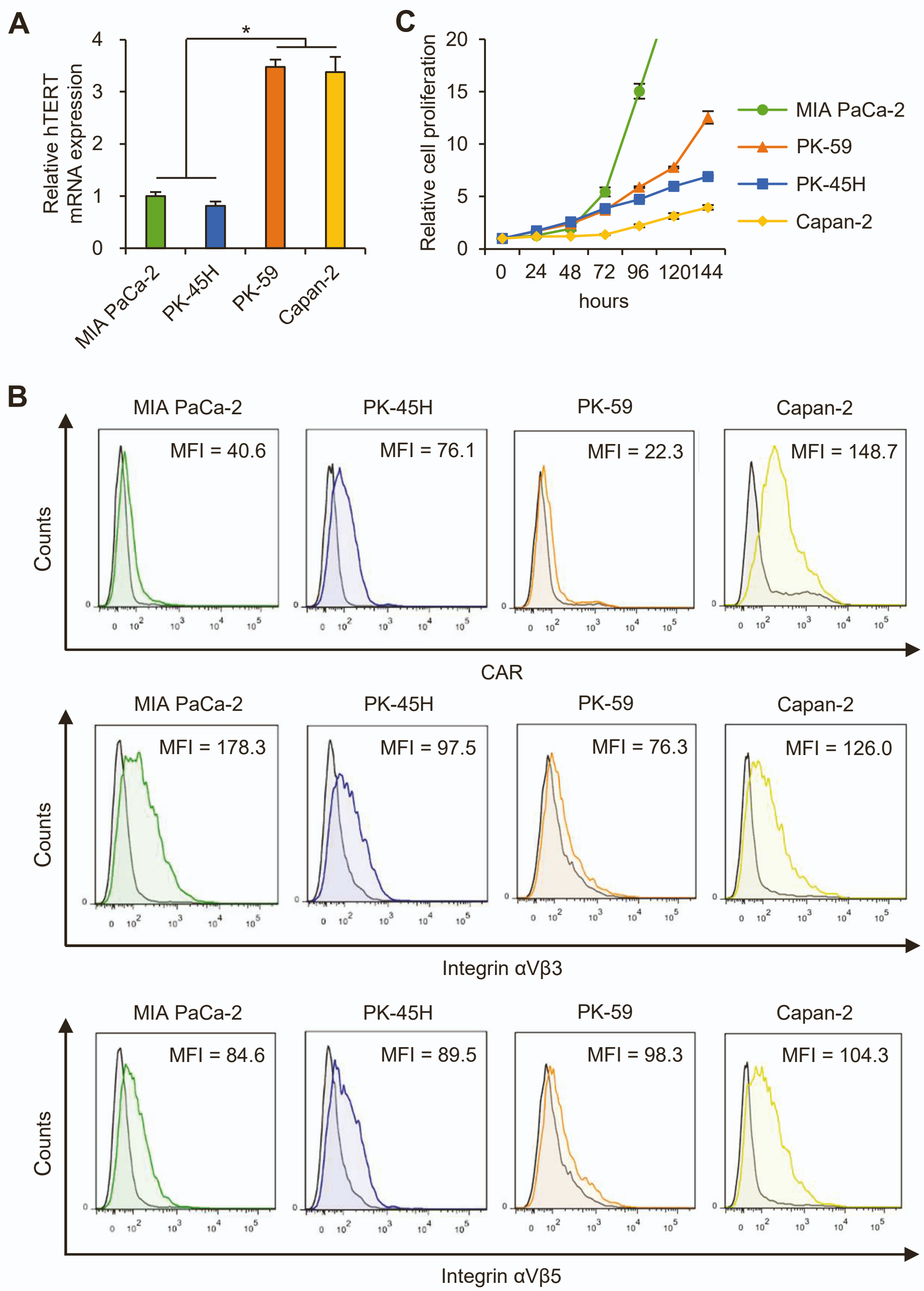

Figure S4. Characterization of expression levels of hTERT mRNA, CAR, and integrins and proliferative rate of human PDAC cells. A, Expression of hTERT mRNA in PDAC cells was analyzed using quantitative RT-PCR. The relative expression of hTERT mRNA in PDAC cells is presented as fold-increase compared with MIA PaCa-2 cells, which was set as 1.0. **B**, Expression of CAR and integrins αvβ3, and αvβ5 on the surface of PDAC cells was assessed by flow cytometry. **C**, Growth curves of PDAC cells as determined using the XTT assay. Cell viability was measured every day and calculated relative to that at 0 h. Data are expressed as mean value (SD) of independent experiment (n = 5).

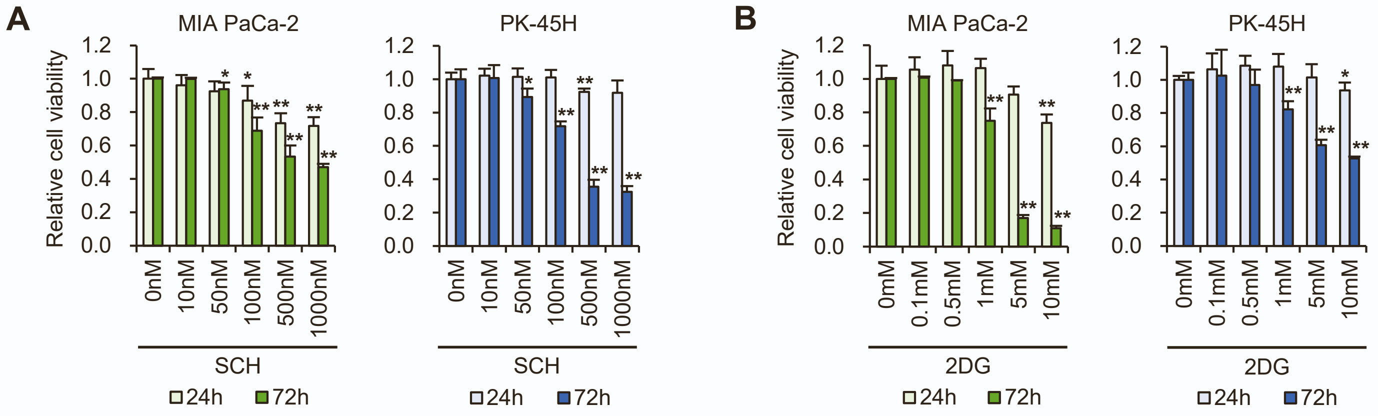

Figure S5. Evaluation of sensitivity of glycolytic PDAC cells to glycolysis inhibitors. A, **B**, MIA PaCa-2 and PK-45H cells were treated with SCH772984 (**A**) or 2DG (**B**) at the indicated dose for 24 and 72 h. Cell viability was quantified using the XTT assay and calculated relative to the mock group. Data are expressed as mean value (SD) of independent experiment (n = 5). The statistical significance of differences between two groups was determined using the Student's *t* test. *, $P < 0.05$; **, $P < 0.01$ (vs 0).

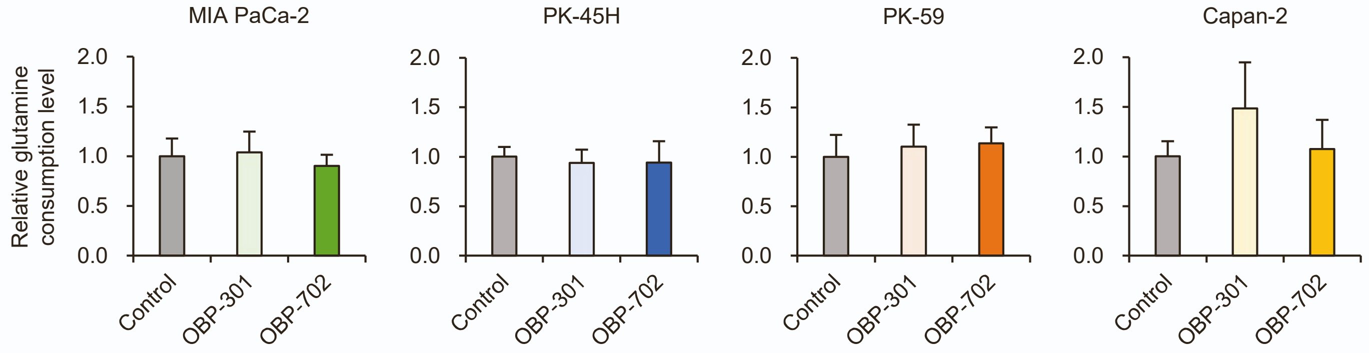

Figure S6. Evaluation of glutamine consumption by human PDAC cells after infection with OBP-301 or OBP-702. The level of glutamine consumption in human PDAC cells was analyzed using a metabolic assay with conditioned medium. The relative level of glutamine consumption by virus-treated cells is presented as fold-increase compared with non-treated control cells, which was set as 1.0. Data are expressed as mean value (SD) of independent experiment (n = 3).

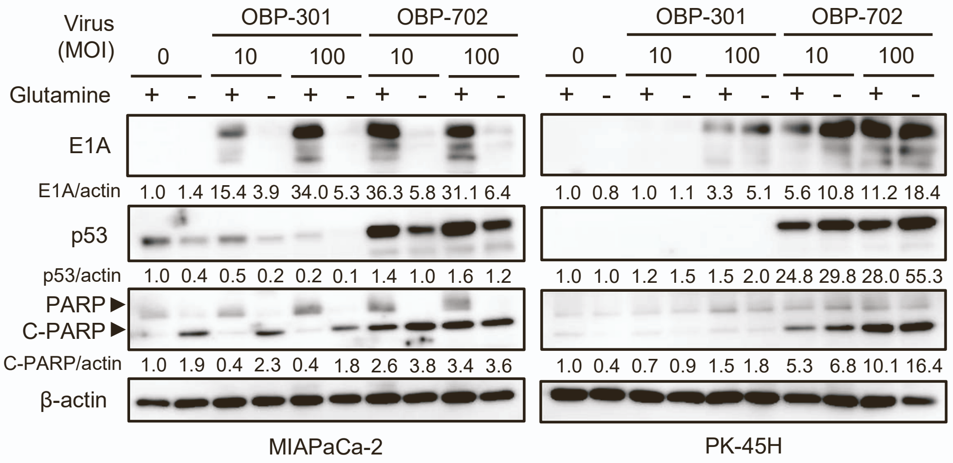

Figure S7. Expression of E1A, p53, and cleaved PARP proteins in glycolytic PDAC cells after infection with OBP-301 or OBP-702 under culture condition with or without glutamine supplementation. Glycolytic PDAC cells were infected with OBP-301 or OBP-702 at the indicated MOIs for 48 h. Cell lysates were subjected to Western blot analysis for E1A, p53, PARP, and cleaved PARP (C-PARP). β-actin was assayed as a loading control. The expression level of each protein was calculated relative to that of mock-treated cells under culture condition with glutamine supplementation.

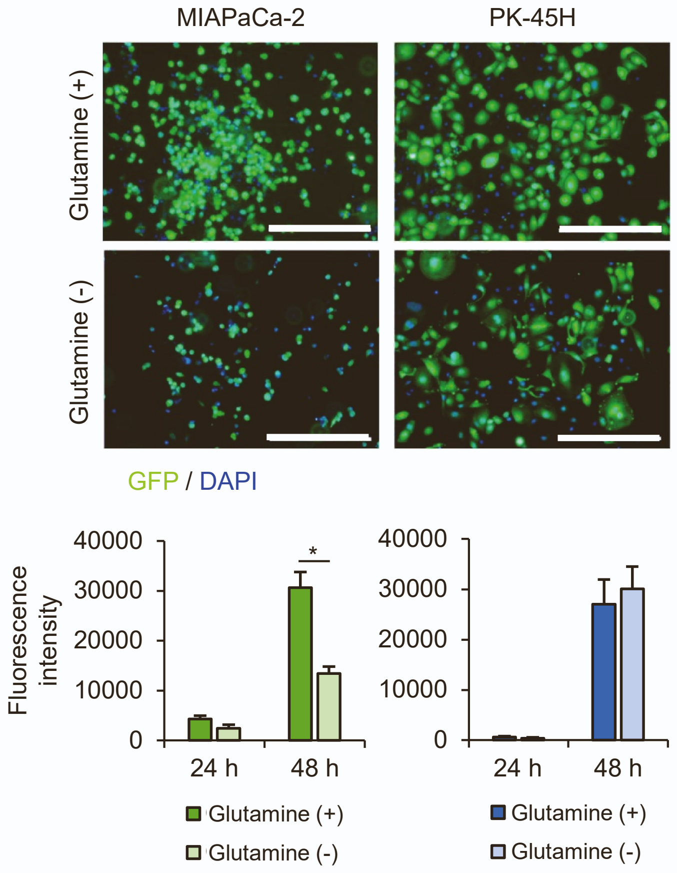

Figure S8. Glutamine deprivation suppresses the OBP-401-mediated GFP expression in glycolytic MIAPaCa-2 cells. MIA PaCa-2 and PK-45 H cells were infected with OBP-401 (100 MOI) for 24 and 48 h under culture conditions with or without glutamine supplementation. Representative photographs of immunocytochemical staining for GFP in each group 48 h after infection. Scale bars, 500 μm. The fluorescence intensity of GFP was analyzed under fluorescence microscopy. Data are expressed as mean value (SD) of independent experiment (n = 3). The statistical significance of differences between two groups was determined using the Student's *t* test. *, $P < 0.05$.

6

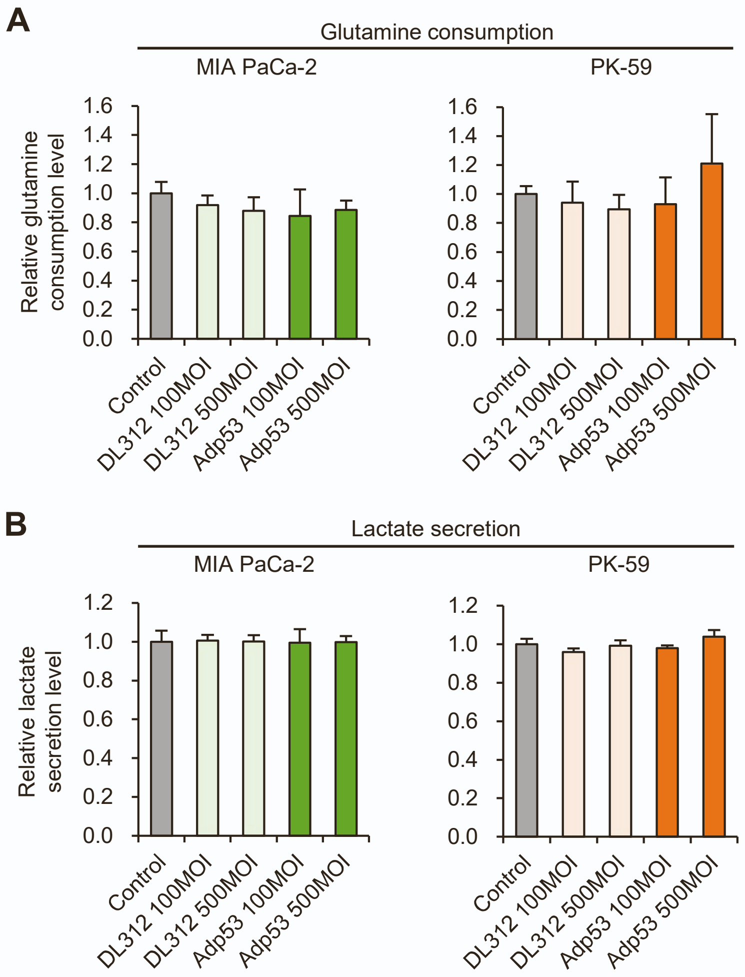

Figure S9. Evaluation of glutamine consumption and lactate secretion by human PDAC cells after infection with DL312 or Ad-p53. MIA PaCa-2 and PK-59 cells were treated with DL312 or Ad-p53 at the indicated MOIs for 72 h. **A**, **B**, Glutamine consumption (**A**) and lactate secretion (**B**) by MIA PaCa-2 and PK-59 cells infected with DL312 or Ad-p53 at the indicated dose for 24 h was analyzed using a metabolic assay. The relative levels of glutamine consumption and lactate secretion by PDAC cells are presented as fold-increase compared with the mock-infected control group, which was set as 1.0. Data are expressed as mean value (SD) of independent experiment (n = 3).

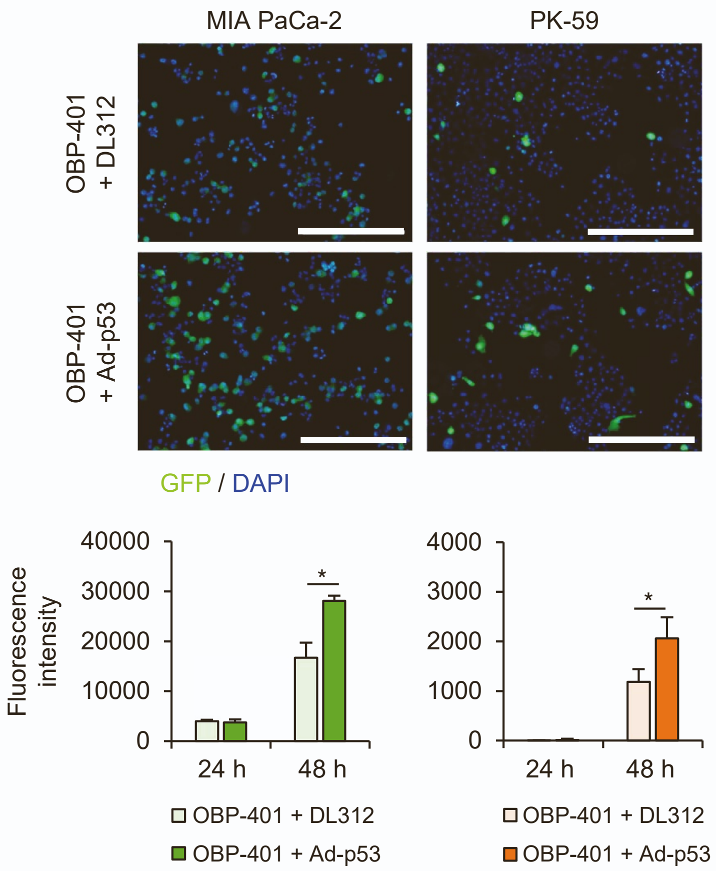

Figure S10. p53 activation enhances sensitivity to oncolytic adenoviruses. MIA PaCa-2 cells were infected with Ad-p53 (100 MOI) or DL312 (100 MOI), followed by infection with OBP-401 (100 MOI) for 24 and 48 h. Representative photographs of immunocytochemical staining for GFP in each group 48 h after infection. Scale bars, 500 μm. The fluorescence intensity of GFP was analyzed under fluorescence microscopy. Data are expressed as mean value (SD) of independent experiment (n = 3). The statistical significance of differences between two groups was determined using the Student's *t* test. *, $P < 0.05$.

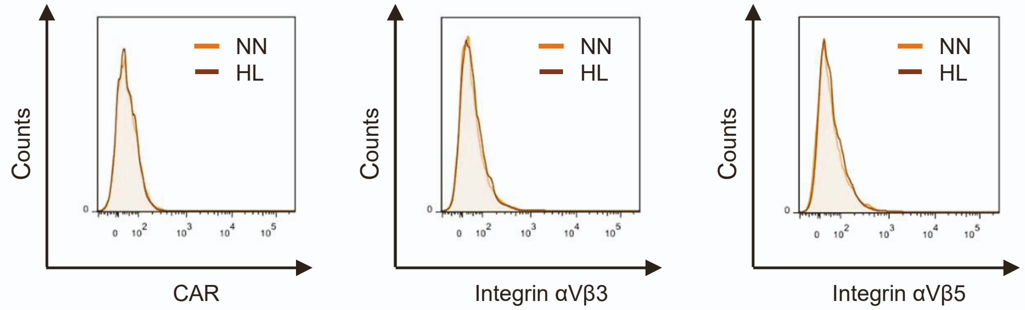

Figure S11. Expression levels of CAR, and integrins in non-glycolytic PK-59 cells under hypoxia and low-glucose culture conditions. Expression of CAR and integrins αvβ3, and αvβ5 on the surface of PK-59 cells under normal (NN) and hypoxia and low-glucose (HL) culture conditions was assessed by flow cytometry.

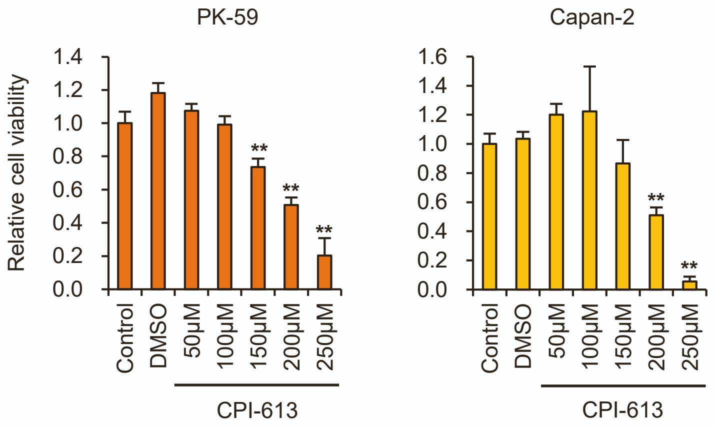

Figure S12. Evaluation of sensitivity of non-glycolytic PDAC cells to mitochondrial metabolism inhibitor. PK-59 and Capan-2 cells were treated with CPI-613 at the indicated dose for 72 h. Cell viability was quantified using the XTT assay and calculated relative to the mock group. Data are expressed as mean value (SD) of independent experiment (n = 5). The statistical significance of differences between two groups was determined using the Student's *t* test. **, $P < 0.01$ (vs control).

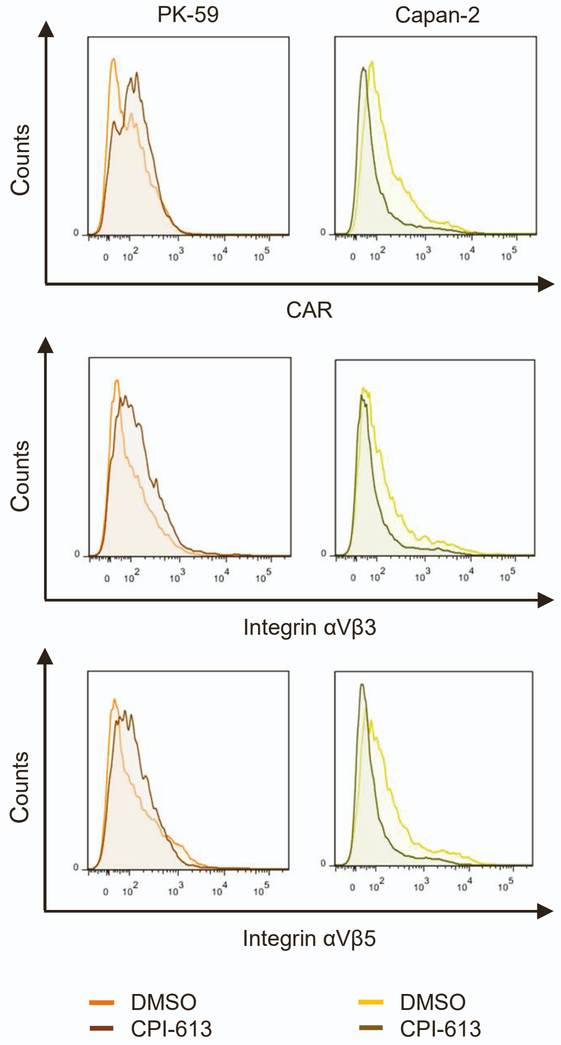

Figure S13. Expression levels of CAR, and integrins in non-glycolytic PK-59 and Capan-2 cells treated with CPI-613. Non-glycolytic PK-59 and Capan-2 cells were treated with CPI-613 for 48 h. Expression of CAR and integrins αvβ3, and αvβ5 was assessed by flow cytometry.

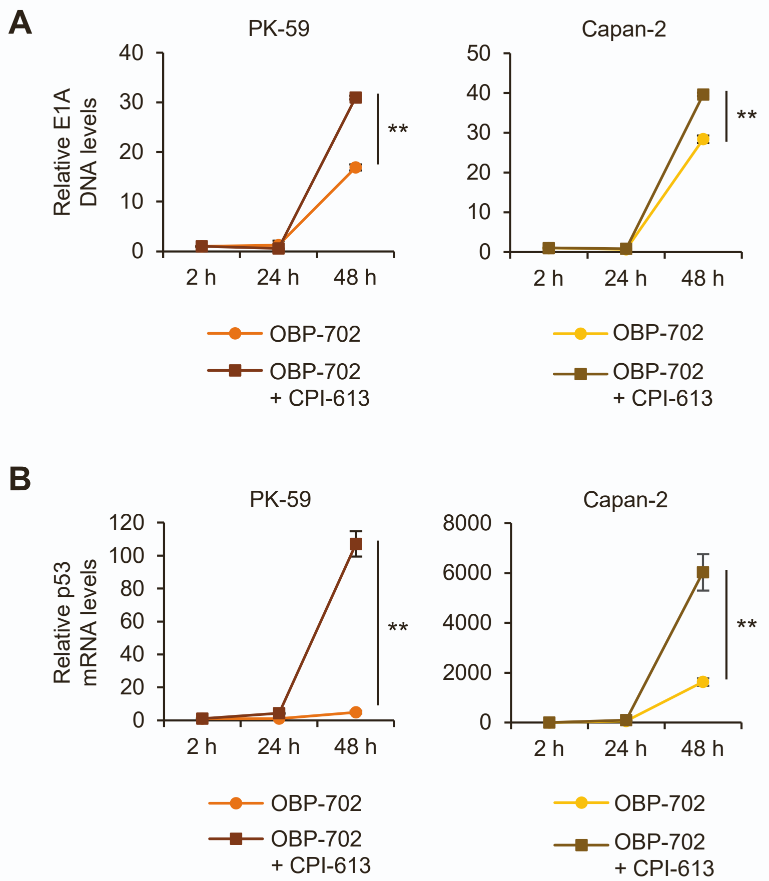

Figure S14. Change in the expression level of adenoviral E1A DNA and p53 mRNA over time in non-glycolytic human PDAC cells treated with OBP-702 and CPI-613. A, B, Copy number of adenoviral E1A DNA (**A**) and expression of p53 mRNA (**B**) were analyzed by real-time RT-PCR. The relative copy number of E1A DNA and relative expression of p53 mRNA are presented as fold-increase compared with 2 h, which was set as 1.0. Data are expressed as mean value (SD) of independent experiment (n = 3). The statistical significance of differences between two groups was determined using the Student's *t* test. **, $P < 0.01$.

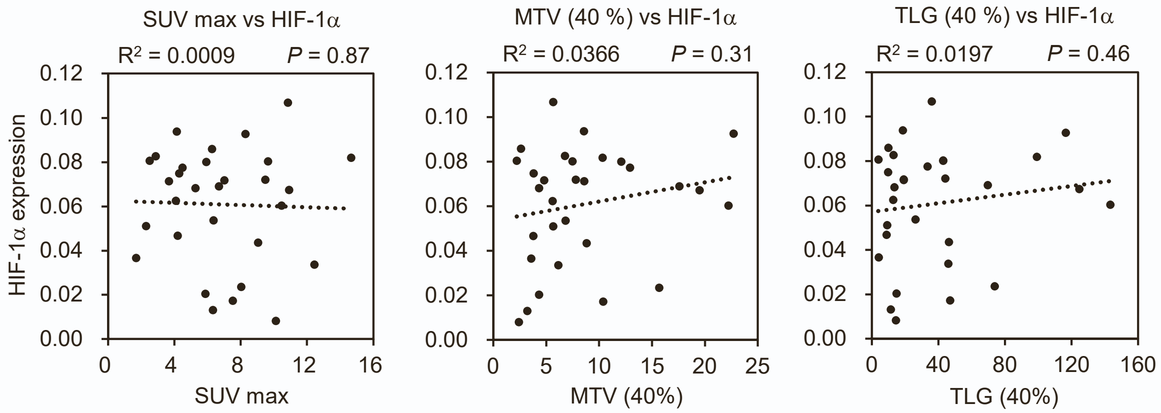

Figure S15. Relationship between PET/CT metabolic parameters and HIF-1α expression in PDAC tumors. Scatter diagram demonstrating the relationship between HIF-1α expression and preoperative SUVmax (left), MTV (40%) (center), and TLG (40 %) (right) values in primary tumors from PDAC patients (n = 30). The statistical significance in the correlation of the scatter plots was determined using Pearson's correlation analysis.
